# Diabetes in pregnancy among indigenous women in Australia, Canada, New Zealand and the United States: a systematic review of the evidence for screening in early pregnancy

**DOI:** 10.1002/dmrr.2389

**Published:** 2013-05-05

**Authors:** Catherine Chamberlain, Bridgette McNamara, Emily D Williams, Daniel Yore, Brian Oldenburg, Jeremy Oats, Sandra Eades

**Affiliations:** 1 International Public Health Unit, Department of Epidemiology and Preventive Medicine, School of Medicine, Nursing and Health Sciences, Monash UniversityPrahan, Victoria, Australia; 2 Baker IDI Heart and Diabetes InstituteMelbourne, Victoria, Australia; 3 Department of Obstetrics and Gynaecology, Melbourne UniversityBurnley, Victoria, Australia

**Keywords:** diabetes, pregnancy, indigenous

## Abstract

Recently proposed international guidelines for screening for gestational diabetes mellitus (GDM) recommend additional screening in early pregnancy for sub-populations at a high risk of type 2 diabetes mellitus (T2DM), such as indigenous women. However, there are criteria that should be met to ensure the benefits outweigh the risks of population-based screening. This review examines the published evidence for early screening for indigenous women as related to these criteria. Any publications were included that referred to diabetes in pregnancy among indigenous women in Australia, Canada, New Zealand and the United States (*n* = 145). The risk of bias was appraised. There is sufficient evidence describing the *epidemiology* of diabetes in pregnancy, demonstrating that it imposes a significant disease burden on indigenous women and their infants at birth and across the lifecourse (*n* = 120 studies). Women with pre-existing T2DM have a higher risk than women who develop GDM during pregnancy. However, there was insufficient evidence to address the remaining five criteria, including the following: *understanding current screening practice and rates* (*n* = 7); *acceptability of GDM screening* (*n* = 0); *efficacy and cost of screening for GDM* (*n* = 3); *availability of effective treatment after diagnosis* (*n* = 6); and *effective systems for follow-up after pregnancy* (*n* = 5). Given the impact of diabetes in pregnancy, particularly undiagnosed T2DM, GDM screening in early pregnancy offers potential benefits for indigenous women. However, researchers, policy makers and clinicians must work together with communities to develop effective strategies for implementation and minimizing the potential risks. Evidence of effective strategies for primary prevention, GDM treatment and follow-up after pregnancy are urgently needed. Copyright © 2013 John Wiley & Sons, Ltd.

## Introduction

Diabetes in pregnancy (DIP) causes serious complications in pregnancy and birth 1 and is an important driver of the type 2 diabetes mellitus (T2DM) epidemic in indigenous populations 2. T2DM is one of the leading causes of death globally 3 and imposes a disproportionately large burden on indigenous people 4. The scale of the public health impact requires a coordinated public health response incorporating a range of primary, secondary and tertiary prevention strategies^5^–^7^, based on evidence to ensure it reduces health disparities and is relevant for policy and practice 8.

Diabetes in pregnancy refers to any diabetes in pregnancy, including gestational diabetes mellitus (GDM), type 2 diabetes mellitus (T2DM) and type 1 diabetes mellitus (T1DM). GDM is defined as ‘any degree of glucose intolerance with onset or first recognition during pregnancy’^9^, although recent international guidelines recommend differentiation between probable T2DM identified early in pregnancy that has not been previously diagnosed and GDM that develops later in pregnancy 10. DIP is associated with poor outcomes for both the mother and her infant during pregnancy, at birth and across the lifecourse 11. The risks for the mother include an increased risk of caesarean section 12, pre-eclampsia and developing T2DM after pregnancy^13^,^14^. The risks for the infant include an increased risk of congenital abnormalities 15, macrosomia 12, neonatal hypoglycaemia 1 and developing T2DM in later life 16, which implicates DIP as having a major compounding effect on the diabetes epidemic 2. Mothers with pre-existing T1DM or T2DM before pregnancy, and their infants, have a higher risk's of complications than those who develop GDM during pregnancy^17^–^19^.

Existing GDM screening guidelines were developed more than 40 years ago by adapting methods for non-pregnant women or to identify those at a higher risk of developing T2DM after pregnancy 12. However, a growing evidence base demonstrating the increased risks of hyperglycaemia in pregnancy 1 and the longer term^2^,^20^,^21^ to both mother and infant, the rising prevalence of GDM 22, and intervention trials demonstrating that there is effective treatment for GDM which improves pregnancy outcomes^11^,^23^,^24^, has led to a revision of these international recommendations^10^,^25^. A key point of debate during this revision process has been whether early pregnancy screening for GDM should be offered universally for all women or selectively to sub-populations at higher risk of T2DM^26^,^27^. Furthermore, the type of tests (particularly in early pregnancy) 28, the timing of tests, what thresholds should be used^29^–^32^, as well as the most effective preventive, treatment and follow-up strategies^26^,^33^,^34^ continue to be discussed^26^,^35^–^38^. However, one area of apparent consensus is that women in sub-populations at high risk of T2DM should be offered screening in early pregnancy at 6–12 weeks of gestation^10^,^25^,^26^,^39^, in addition to screening at 24–28 weeks of gestation as is currently recommended.

Screening is a secondary prevention strategy where the aim is to reduce the burden of disease in the community through early detection of disease, providing an opportunity for therapeutic intervention and improved health outcomes^40^,^41^. There are, however, long established criteria^40^–^42^ that should be met before introducing population-based screening, to ensure that the benefits outweigh any risks, inconvenience and costs (Table 1).

**Table 1:** Criteria for population-based screening

Criterion	Evidence required
Epidemiology of disease	Prevalence and natural history are understood, and the condition poses a significant disease burden
Current screening practice and rates	Barriers and facilitators are understood
Acceptability	Women's preferences and values are understood
Efficacy and cost	Sensitive and specific cost-effective screening tests are available
Effective treatment	Available and accessible after diagnosis
Reliable follow-up systems	In place for those diagnosed at risk

There are a number of potential benefits to offering early screening for GDM, which may be particularly important for indigenous women who have a high risk of T2DM. Primarily, the early detection and treatment of DIP has been shown to reduce the associated health risks in pregnancy and birth among non-indigenous women^11^,^23^,^24^,^43^,^44^. Furthermore, pregnancy offers a ‘window of opportunity’ for health interventions, as predominantly young healthy pregnant women have frequent scheduled contacts with health-care providers. They are often highly motivated to adapt their behaviour to improve the health of their infant^45^,^46^, with any effective lifestyle interventions potentially benefitting the whole family^47^,^48^. In addition, pregnancy mimics a ‘natural stress test’ 49 for insulin resistance as a result of naturally occurring placental hormones^50^,^51^, offering a unique opportunity for detecting the disease at an earlier stage in the natural history of this metabolic disorder.

However, there also is the potential for harm to result from the introduction of early pregnancy GDM screening. The increased diagnosis of any medical condition in a generally healthy population can be associated with an increased psychological stress 52. This is particularly the case during pregnancy as women are concerned about the health of their infant^53^–^56^. Although recent studies among non-indigenous women suggest that this is not necessarily the case with a GDM diagnosis^11^,^54^, indigenous women may experience unique stressors 57, including dislocation from families if required to move from a remote community to a regional centre for obstetric care. There is potential for any intervention during pregnancy to interfere with the normal processes and initiate a ‘cascade of medical interventions’ 58, such as induction of labour, caesarean section and artificial infant feeding^23^,^35^,^59^. There are also risks with selectively applying a preventive strategy to sub-populations, including ‘labelling’ 60, which can exacerbate existing social stigma, as well as internalised racism and negative self-esteem^61^,^62^.

In addition to specific risks, the evidence in relation to the specific population-based screening criteria (Table 1) is likely to be different for indigenous women 63. Indigenous people continue to experience poorer health than other people living in the same country 63, and the epidemiological patterns of diabetic disease are markedly different, implicating DIP as a major contributing factor 2. Efforts to reduce these health inequalities have led to an identified need to assess the potential differential impact of interventions^8^,^64^. Compounding this is evidence that indigenous people experience reduced access to treatment for diabetic complications^65^–^69^ and DIP 70. Although the efficacy of screening tests and pharmaceutical treatments is based on biological evidence that is likely to be similar for all population groups, the effectiveness may differ according to the setting and population (context) in which an intervention is delivered 71, further supporting the need to examine indigenous-specific evidence.

To our knowledge, this is the first review to examine the evidence in relation to the recent International Association of Diabetes and Pregnancy Study Groups recommendations for early GDM screening in indigenous populations. This systematic review aims to assess the level of evidence for early screening for GDM among indigenous women in Australia, Canada, New Zealand and the United States.

## Materials and methods

The methodology for this review has been described in detail elsewhere 72.

### Inclusion criteria

All publications, with the exception of abstracts, that focused on DIP among indigenous women in Australia, Canada, New Zealand or the United States in the title or abstract were included. We excluded publications that focused on diabetes among children or adults where there was only a very brief reference to DIP. This broad inclusion criterion was used to ensure all study designs could be examined and included if they contained any qualitative or quantitative data relevant to the population-based screening criteria^40^,^42^. It was decided to focus on published studies concerning indigenous women in these four countries because they have been compared in other related reviews^73^,^74^ as they share similar experiences associated with colonisation, marginalisation, institutionalisation, poverty, a rapid transition from a traditional to a westernised lifestyle and an increased risk of diabetes.

### Search method for identification of studies

We searched the Cochrane Database for Systematic Reviews (1995 to July 2012), Medline (1950 to July 2012), Embase (1949 to July 2012), CINAHL (1937 to July 2012) and PsychINFO (1905 to July 2012) to identify published literature. A comprehensive key word and MeSH heading search strategy for related terms associated with ‘pregnancy’ and ‘diabetes’ and ‘indigenous’ was used 72. Appendix A shows the full search strategy used for Embase, with adapted MeSH terms for other databases. No language restrictions were applied.

### Data collection

The abstracts of all search results were reviewed by two authors (C. C. and E. W.) to determine those potentially meeting the inclusion criteria. The full texts of these publications were then reviewed by one author (C. C.), with a random selection (10%) independently reviewed by another author (D. Y.) for validation. Data from publications meeting inclusion criteria were extracted by one author (C. C.) and a random selection of 10% independently extracted by another author (D. Y.) for validation. Data items included the population description, study details and the main findings relevant to the population-based screening criteria 40.

#### Appraisal of external validity (generalisability)

To assess the potential generalisability of the study, data were extracted on the basis of whether the study was conducted in a population that was remote, rural, urban or mixed, and whether the data source used was population, community or clinic based.

#### Appraisal of internal validity (risk of bias)

Intervention studies, measurement studies and systematic reviews were assessed using appraisal tools developed by the Centre for Evidence Based Medicine^75^–^77^. Qualitative studies were appraised using tools developed by the Australian Department of General Practice and other local experts^78^,^79^. There was no standard tool for appraising quantitative descriptive observational studies; therefore, one was adapted for this review from the *Strengthening the Reporting of Observational Studies in Epidemiology* statement 80 and other published tools developed for reviewing epidemiological studies^81^,^82^.

### Data synthesis

The study results were synthesised under each of the relevant screening criteria. General ‘evidence statements’ were generated from the publications, and a ‘level of evidence’ reported according to pre-specified criteria based on an adapted^83^,^84^ GRADE tool 72 (Table 1). The ‘level of evidence’ related only to the internal validity (risk of bias) appraisal of the study from which the evidence statement was generated and does not bear any relationship with whether the screening criteria are met. The country in which the study was conducted was reported as an indication of generalisability (external validity) for each evidence statement. An assessment of whether the evidence was *sufficient* or *insufficient* to meet each criterion was made by the authors, taking into the consideration the ‘level of the evidence’ (risk of bias) of the studies from which the evidence statements were generated and the scope of the issues addressed by the evidence statements (Table 2).

**Table 2:** Classification of the level of evidence for included studies

Symbol	Level of evidence	Risk of bias criteria
H	High	One or more study with low risk of bias
M	Moderate	One or more studies appraised with moderate risk of bias
L	Low	One or more studies were appraised with high risk of bias
VL	Very low	The publications were not in a format that allowed appraisal of the effect estimate (e.g. opinion piece)

## Results

**Figure 1:**
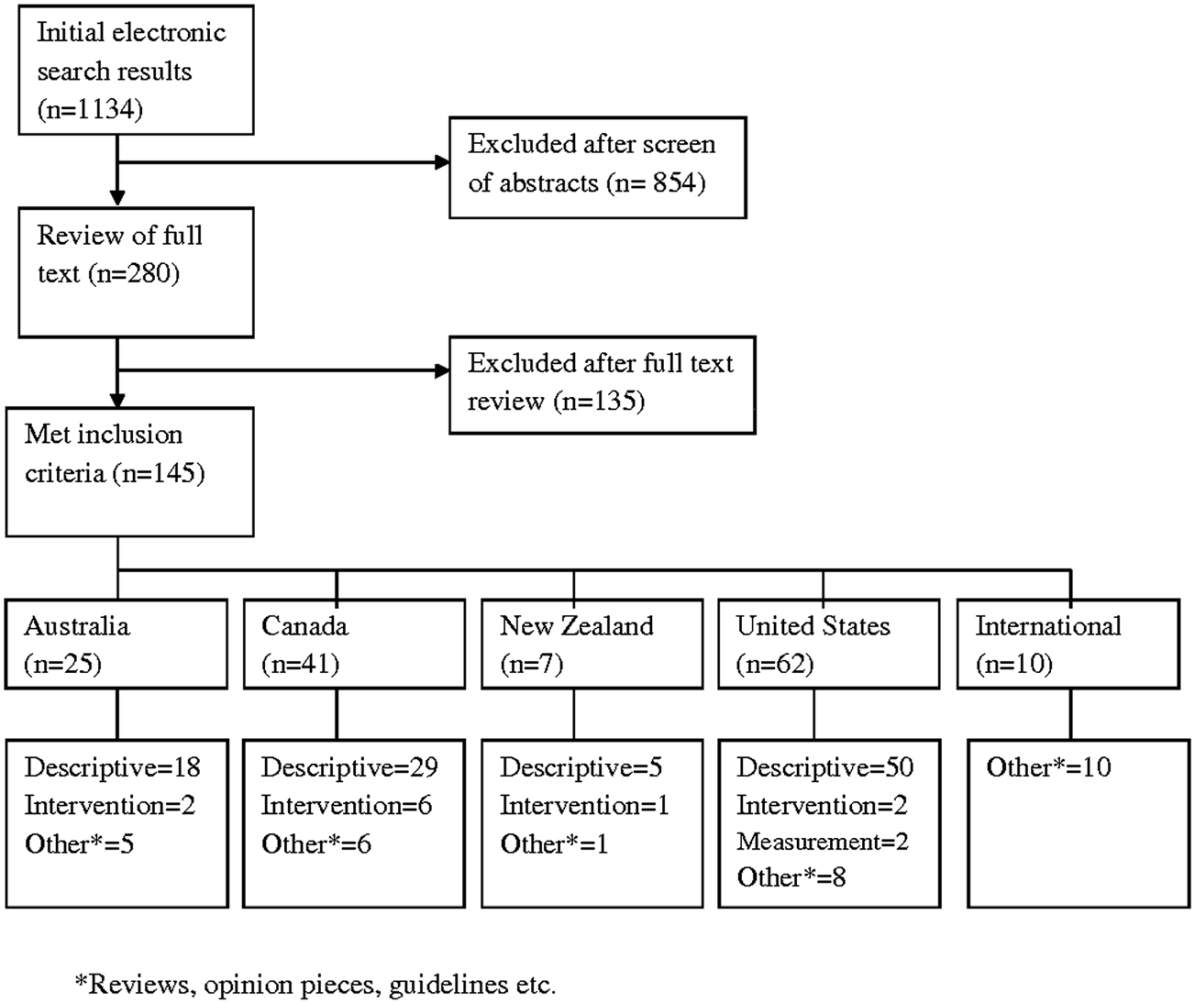
Flow chart for included studies

The initial search using terms related to ‘diabetes’ and ‘pregnancy’ yielded over 40 000 results, which was reduced to 1134 when the ‘indigenous’ terms were applied as a filter. The abstracts of these 1134 publications were screened, and 854 abstracts were excluded as they were clearly unrelated to DIP among indigenous women in Australia, Canada, New Zealand or the United States. The full text of 280 publications was reviewed, and an additional 135 were excluded. The most common reason for exclusion was that the abstract discussed diabetes *and* pregnancy as separate factors, rather than discussing diabetes *in* pregnancy. A total of 145 publications were included, and a detailed table of the study characteristics and risk of bias appraisal is available on request. Validation checks showed that there was good agreement (>96%) with assessment of whether studies met inclusion criteria and high levels of agreement (>80%) for the risk of bias appraisals (Figure 1).

### Description of included publications

The majority (96/145 or 66%) of included studies were quantitative descriptive studies, with a range of study designs, including findings from over 12 million people. There were only six qualitative descriptive studies including 140 participants. Eleven (8%) publications (7073 participants) described or evaluated interventions, but only one of these studies was randomised, and therefore, 12 of these studies were appraised as having a high risk of bias. Two measurement studies (308 participants) evaluated screening test efficacy. A total of 30 (21%) other publications did not report primary research, including 23 reviews and seven opinions, commentaries, editorials or guidelines.

### Risk of bias (internal validity)

**Figure 2:**
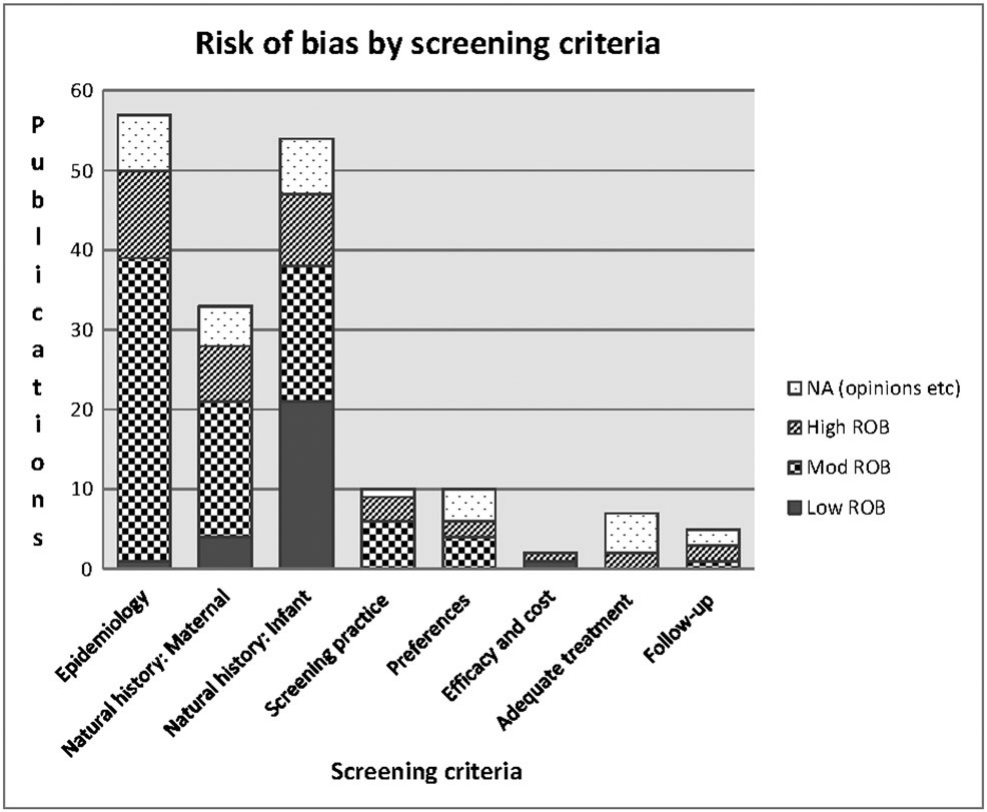
Number of studies graded as high, moderate, low or not appraisable under each of the population-based screening criteria

Only 22/96 (23%) of the quantitative descriptive studies met all the criteria for ‘low risk of bias’ in this review; the majority (*n* = 16) of which were generated from a longitudinal study among Pima and Papago Indian communities in the United States. The major risk of bias identified in the quantitative studies was outcome assessment bias, as diabetes was often indirectly measured using medical records or hospital registers (49/96 or 51%). The majority of quantitative descriptive studies (64/96 or 67%) adequately described the diagnostic criteria used for the identification of DIP or GDM. However, the lack of diagnostic criteria in 33% of studies was a major limitation given the variability of diagnostic and screening criteria used in practice. Selection bias is a complex issue in reviews involving identification of ethnicity. There were 70/96 (73%) that included whole or consecutive samples and were therefore coded as low risk of selection bias because they were representative of the group described (if the participation rate was high). Only one study in Australia attempted to quantify the identification rate 85 and estimated that approximately 20% of Aboriginal and Torres Strait Islander people were not identified in the sample. In Canada and the United States, where indigenous people are registered according to proportion of indigenous heritage, the inclusion criteria was frequently limited to registered people with over 50% heritage. The risk of bias for studies according to each of the screening criteria is summarised in Figure 2.

### External validity (generalisability)

**Figure 3:**
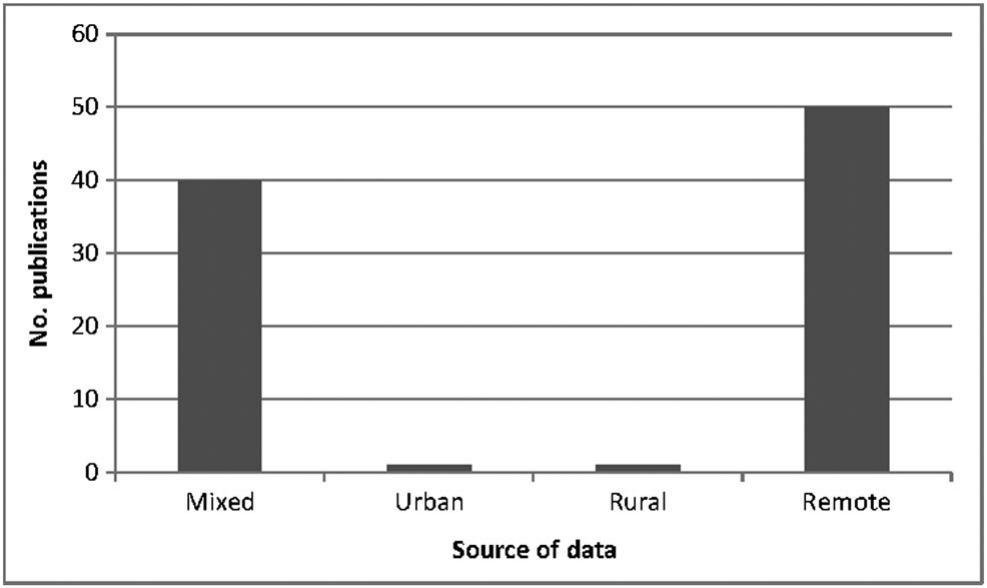
Number of descriptive studies conducted in mixed, urban, rural and remote populations (generalisability)

Only two descriptive studies were conducted among indigenous women living in urban areas, compared with 50 conducted in remote communities (Figure 3). Although the 40 descriptive studies in mixed populations were potentially generalisable to a wider population, many of the mixed population studies were appraised as having a high risk of outcome assessment bias due to their reliance on database reporting.

### Main findings and strength of evidence for each of the screening criteria 40

The results are summarised in Table 3. The first column lists the screening criteria, with the number of studies addressing that criterion in parentheses below, and some papers addressing more than one criterion. The first screening criterion (epidemiology) is disaggregated into seven sub-categories as most of the included studies (70%) addressed this factor. The second column lists ‘evidence statements’ generated, with the third column summarising the number of studies appraised at each ‘level of evidence’ (high [H], medium [M], low [L] and very low [VL]), with the respective references in parentheses. The final column states the country where the studies generating those evidence statements were conducted.

1. Epidemiology (prevalence and natural history)

**Table 3:** Summary of evidence for population-based screening for diabetes in early pregnancy among indigenous women in Australia, Canada, New Zealand and the United States

Screening criterion (no. publications)	Evidence statement	Quality of evidence for each statement and study references (H, high; M, moderate; L, low; VL, very low)	Country
1. Prevalence (*n* = 49)	Higher risk of undiagnosed T2DM in pregnancy and GDM	H 86	Aus, Can, NZ, US, Int
		M^14^,^15^,^18^,^21^,^87^–^115^,^123^,^127^	
		L^13^,^116^–^122^,220	
		VL^126^,^145^,^169^,221,222	
Prevalence (trends) (*n* = 7)	Prevalence of GDM and T2DM in pregnancy is increasing	M^21^,^125^,^167^	Aus, Can, US, Int
		L [223,224]	
		VL [225,226]	
Natural history: risk for maternal development of DIP (*n* = 10)	Maternal birth-weight low and high (u-shaped association)	H 156	US
		M [227]	
	Obesity	M^99^,^123^	Can, US Aus
		L 122	
	Genetic variants	H [228]	US
	Thrifty genotype theory	VL^2^,^145^,^161^	Can, Int
	Thrifty phenotype theory	VL 160	US
Natural history: risk to woman during pregnancy and birth (*n* = 12)	Adverse birth outcomes (e.g. caesarean section and shoulder dystocia)	H 140	Aus, Can, NZ, US
		M^92^–^95^,^111^,^113^,^129^,^133^	
	Increased risk of hospitalisation, associated with acute renal disease	M 136	Can, US
		L^118^,^137^	
Natural history: maternal progression to T2DM (*n* = 8)	Non-pregnant women with impaired glucose intolerance have higher risk of T2DM than pregnant women with impaired glucose tolerance	H [229]	US
		L 13	
	Increased risk of progressing from GDM to T2DM	M^14^,^21^,^144^	Can, US
		L 13	
		VL 145	
	Faster progression from GDM to T2DM	M^96^,^112^	Aus, Can
		L 13	
	Progress from GDM to T2DM at a younger age	M 96	Aus
Natural history: risk to infant in pregnancy and birth (*n* = 21)	Increased risk of congenital abnormalities	H 131	Aus, US
		M^15^,^108^,^132^	
		VL 138	
	Increased risk of macrosomia	H^128^,^139^,^140^	Aus, Can, NZ, US
		M^109^,^130^,^135^,^141^–^143^	
		L 128,230,231	
	Increased risk NICU admissions or poor birth outcomes	M^94^,^113^,232	Aus, Can
		L [233]	
		VL [234]	
Natural history: long terms risks to infant (*n* = 31)	Increased risk of obesity	H^146^–^148^	Can, US
		L 128	
		VL 149	
	Increased risk of glucose intolerance	H^148^,^150^–^152^, M 153	US
	Increased risk of GDM and T2DM	H^16^,^148^,^154^–^157^	Can, US, Int
		M^16^,^127^,^135^,^158^,^165^,^166^^,^235	
		L^128^,^163^,^164^	
		VL^2^,^159^–^162^	
	Increased risk renal disease	H [236,237]	US
2. Current screening practice and rates (*n* = 7)	GDM screening practice and rates is variable	M^88^,^89^,^167^^,^238	Aus, Can, NZ, US
		L^13^,^168^	
		VL 169	
	Highest risk women (e.g. obese women) may be less likely to be screened	M^88^,^89^	NZ, US
3. Preferences or values (*n* = 8)	Suggest resources be culturally adapted, programs provide blood sugar data and emphasize opportunity to save money with health diet	M 176	Can
	Prefer greater community involvement (especially midwives and elders) and recognise importance of family ties and cultural values	M 176	Can
		VL^171^,^177^	
	Prefer group sessions and less direct advice (e.g. story-telling)	VL 171	Can
	Concern about weight gain in pregnancy but many barriers	VL 178	Can
	Many mixed understandings of risk and causes of DIP	M 172	Aus, Can, US
		L^173^,^174^	
	Diet (grandmothers), exercise and stress (mothers) cause DIP	L 175	Can
4. Efficacy and cost (*n* = 3)	Screening more sensitive than risk factor analysis alone	H 180	US
	One-step WHO method more sensitive than two-step NDDG method	L 179	US
	HBA_1C_ tests not appropriate screening tool among indigenous women	L 181	Int
5. Adequate treatment pathways (*n* = 6)	Integrated community care may improve self-monitoring	L 186	Aus
	Standards for diagnosis and treatment	VL 182	US
	Early screening needed to reduce risk of GDM to mother and baby	VL^144^,^183^,^184^	Aus, Can
	Insulin pumps may improve glycaemic control	L 185	NZ
6. Follow-up after pregnancy (*n* = 5)	Low rates of follow-up screening for T2DM after pregnancy for women diagnosed with GDM	M 14	Can, NZ, US
		L 13	
		VL 188	
	Registers may improve follow-up	VL 189	Can
	High rates of glucose intolerance in women with DIP followed up after pregnancy	L 188	NZ

DIP, diabetes in pregnancy; GDM, gestational diabetes mellitus; NDDG, National Diabetes Data Group; NICU, neonatal intensive care unit; T2DM, type 2 diabetes mellitus; WHO, World Health Organisation.

A large amount of evidence (over 70%), much of which was appraised as a moderate to high level, described the *epidemiology* of DIP among indigenous women and their infants. Indigenous women generally showed a significantly higher risk of GDM and T2DM^13^–^15^,^18^,^21^,^85^–^125^, often occurring at a younger age 126, compared with other women in the same country 127. The main risk factor for developing T2DM was obesity 122, with over 50% of DIP among Native Americans attributed to obesity 123. DIP was clearly associated with a range of adverse consequences in the short term for both women and their infants, and women diagnosed with pre-existing T2DM demonstrated the highest risk^15^,^108^,^128^–^132^. These risks included caesarean section 133, shoulder dystocia, increased hospitalisation, congenital abnormalities, macrosomia^134^,^135^, neonatal intensive care admissions and hypoglycaemia^15^,^92^–^95^,^108^,^109^,^111^,^113^,^118^,^128^–^132^,^136^–^143^. In the longer term, indigenous women were shown to have a higher risk of developing T2DM after pregnancy^13^,^14^,^21^,^96^,^112^,^144^,^145^. Despite the search strategy not being designed for capturing all research about the risk of DIP to infants, 21% (31/145) of included studies demonstrated that infants born to women with DIP have a higher risk of developing obesity, hyperglycaemia, T2DM and renal disease^16^,^128^,^146^–^165^. One modelling study estimated that approximately 19–30% of T2DM among Canadian First Nations people is attributable to in utero exposure to GDM, compared with only 6% among the rest of the Canadian population 166. An impaired ‘acute insulin response’ among children exposed to DIP^150^,^151^ was proposed as a possible mechanism for the apparent intergenerational effect.

2. Current screening practice and rates

Seven studies described *current screening practice and rates*^13^,^88^,^89^,^167^–^170^. They suggested there has been little consistency in GDM screening rates among indigenous women, with some studies reporting less than 50% of women receiving screening in pregnancy^13^,^88^,^89^. One study in a remote island community in Australia reported 99.5% of women were screened during pregnancy 167; however, a review of other services in remote areas reported screening rates ranging from 3% to 78% 170. Two studies reported that women at high risk of GDM due to obesity were even less likely to receive screening than women of normal weight^88^,^89^.

3. Acceptability

No publications reported the acceptability of GDM screening for indigenous women, and only eight publications reported the *preferences and values* of indigenous women related to DIP more generally^171^–^178^. One opinion piece outlined from an indigenous perspective why a particular intervention that had intended to reduce GDM rates had not been effective 171, and another argued for the importance of looking at the ‘root cause’ of behavioural risk factors and engaging with indigenous communities to become advocates for social change 177. Three qualitative studies appraised as providing a moderate level of evidence described mixed levels of understanding of the risks and causes of DIP among both indigenous women and their care providers 172; outlined the importance of family ties, preserving cultural values and adapting resources, and ensuring access to blood sugar data as a means of control 176; and described the perceptions of weight gain and the challenges in losing it after pregnancy 178.

4. Screening test efficacy and cost

Two descriptive studies reported GDM screening efficacy at 24–28 weeks among indigenous women^179^,^180^. However, no studies reported the efficacy for GDM screening early in pregnancy in this population. One study, appraised as providing a high level of evidence, demonstrated that universal screening for GDM is significantly more sensitive than risk factor analysis alone 180. The second study suggested that the ‘one-step’ 2-h 75 g oral glucose tolerance test was more sensitive than the ‘two-step’ O'Sullivan criteria 179. One review suggested that the use of the HbA_1c_ test is not appropriate as a diagnostic or screening test among non-European populations, as it has specificity and higher variability as a result of biological and genetic factors 181.

5. Effective treatment available after diagnosis

Six publications related to treatment strategies for DIP among indigenous women were identified, all of which were appraised as providing a low to very low level of evidence. Four publications were opinion pieces about the recommended treatment regimes for DIP^144^,^182^–^184^. One case–control study suggests insulin pumps may provide better glycaemic control; however, there were increased neonatal intensive care unit admissions in the intervention group, although they did have higher baseline insulin requirements 185. Another intervention study, with no control group, indicated that the development of an integrated care programme in a community-controlled health service improved monitoring for women 186. One study, appraised as providing a moderate level of evidence, reported higher rates of macrosomia for indigenous infants compared with non-indigenous infants, despite controlling for body mass index and GDM, and the authors suggested that the difference may be due to different treatment strategies for indigenous women 187.

6. Follow-up after pregnancy for women at risk of T2DM

Despite clear evidence that indigenous women have a higher risk of developing T2DM after pregnancy^14^,^21^,^144^,^188^, only five publications discussed *follow-up* after pregnancy. Three studies reported low rates (<40%) of follow-up screening for T2DM after pregnancy for indigenous women^13^,^14^,^188^. One project report described the development of a register designed to improve follow-up 189 but did not report whether this strategy was effective.

Other publications (including primary prevention)

One randomised controlled trial demonstrated a significant increase in the rate of knowledge of diabetes and obesity in the intervention group 190. Three studies, appraised as providing a low level of evidence, reported no effect from exercise or nutritional interventions^191^,^192^ and reported significant barriers to recruiting women to participate in the intervention 193. No rigorous evaluations of strategies to increase and support breastfeeding for indigenous women with DIP were found, despite solid evidence identifying breastfeeding as having a protective effect for infants against the development of T2DM^16^,^194^ and its feasibility for implementation in indigenous communities 195. Furthermore, although the importance of addressing broader environmental issues was proposed in four published opinion pieces^177^,^196^–^198^, there were no published evaluations of any environmental strategies to reduce DIP.

## Discussion

This article has reviewed published studies related to DIP among indigenous women in Australia, Canada, New Zealand and the United States to evaluate the level of evidence available to address the criteria for population-based screening in early pregnancy. There was sufficient evidence describing the *epidemiology* of DIP, which clearly demonstrates that indigenous women have a higher risk of DIP, particularly T2DM, compared with other women in the same country. This has serious health consequences for both women and their infants in pregnancy, at birth and across the lifecourse. There was good evidence to suggest indigenous women meet the criteria for a population at ‘high risk’ of T2DM^26^,^39^, which is associated with a higher risk than GDM in pregnancy for women and their infants^15^,^108^,^128^–^132^. Early detection of DIP therefore offers potential benefits for women, their infants and the broader community, if effective interventions are provided.

However, there was insufficient evidence to determine that the remaining five criteria are met for introducing population-based screening for GDM in early pregnancy among indigenous women and to assess whether the potential benefits outweigh the risks. There was insufficient evidence to demonstrate that *current screening practices* are effective, with variable rates reported, and some evidence suggesting that women with the highest risk due to obesity may be even less likely to be screened. There was insufficient evidence to understand whether the proposed changes are *acceptable* to indigenous women or their preferences and values in relation to screening in early pregnancy. That no evidence exists concerning the acceptability of DIP screening options among indigenous women is a critical consideration because acceptability affects the overall sensitivity and effectiveness of screening when offered at a population level, irrespective of test efficacy^199^. Furthermore, there were no studies found evaluating the potential risks of early GDM diagnosis on indigenous women, including psychological stress or negative self-esteem, social dislocation or physical outcomes as a result of increased intervention 57. There was insufficient evidence to demonstrate which screening test is the most *efficacious and cost effective* in early pregnancy and that *effective treatment options* are available. There is a need to demonstrate *effectiveness* of strategies to improve screening and treatment in real-world settings, particularly as it has been suggested that differential rates of infant macrosomia may have been due to inequitable treatment for First Nations Canadian women diagnosed with GDM^70^,^187^. There was insufficient evidence that demonstrates an effective system to ensure *follow-up after pregnancy* for women diagnosed with GDM who have a high risk of developing T2DM. Studies among non-indigenous people suggest simple reminders may be effective^200^. In addition, there was very limited research conducted among indigenous women living in urban areas, despite this being where the majority of indigenous women now live^201^.

That only two publications written from an indigenous perspective were identified by this review represents an inherent perspective bias in the evidence-base for this topic area. The low rate of participation of indigenous people in higher education and research is likely to be a major reason for this paucity^202^. Another may be that public health research strives to portray an image of objectivity and rarely recognises the subjectivity of the standpoint of the people developing the research agendas or programmes^64^,^203^. However, diabetes is grounded in a complex web of social, historical and personal factors, and understanding perspectives and recognising relativity are critical to understanding and addressing this major public health issue^204^. In addition, the notion of ‘race’ itself is often used as a crude proxy marker for presumed biological and social differences, and therefore, research in this area would be better informed with social science input^203^.

There are several limitations to this review. Firstly, the heterogeneity of the study designs prohibited meta-analysis, so study findings were not weighted, and there were no analysis of the combined effect or sensitivity analysis for the effect of multiple confounders identified, including the risk of bias of included studies. Secondly, the data extraction and risk of bias appraisal was primarily conducted by one reviewer, and we were unable to calculate kappa scores as the high rates of expected concordance required a larger sample size than was feasible within the resource constraints of this review. Thirdly, the Australian population-based screening criteria used may differ from criteria used elsewhere 42. Fourth, it is likely that we have not captured all studies that have included indigenous women in a general sample, and some of the evidence from other populations may be relevant. However, this is likely to have been captured and assessed in recent similar reviews among the general population 26, and our reviews serves to shine the spotlight on the evidence for this sub-population. Finally, only published literature was used in this review, and a significant amount of grey literature was excluded. Inclusion of grey literature would have biased the search results in favour of the country the authors of the review originated (Australia), as familiarity and access to unpublished information was not as readily available across the other three countries.

Our finding that there is the limited evidence for GDM screening among indigenous women is similar to a review examining the evidence-base among non-indigenous women 26, although our analysis has shown that the quality and quantity of evidence for indigenous women is significantly more limited. This review makes similar conclusions to other major studies among non-indigenous women with regard to the risks of DIP 12, low rates of screening during and after pregnancy^205^,^206^, and the challenges with nutritional and exercise interventions to prevent or reduce GDM^207^–^209^. A review of research gaps for the general community also identified a need for more research into effective treatment and management strategies for women with DIP and for improved post-pregnancy follow-up^210^. This review reinforces the findings of other reviews, which conclude that indigenous women have a higher risk of DIP, particularly T2DM, at a younger age 2, and this will significantly increase following adoption of the proposed recommendations^43^,^211^–^216^. Although this review did not include studies with a sole focus on long-term risk to offspring of mothers with DIP, over 20% of included studies identified significant risks, which is consistent with findings of a recent review of the origins of cardiometabolic disease among indigenous populations^217^. Our findings are similar to another review that concluded there is a paucity of high quality interventions for T2DM in indigenous populations 73. This is of particular concern as several opinion pieces in this review argued for broader environmental approaches to reduce the burden of diabetes^177^,^196^–^198^, and it appears there is limited primary prevention interventions in diabetes research more generally. However, one study reported that an intervention that supported breastfeeding^218^ and promoted reduced soft-drink consumption was both feasible and effective in reducing obesity among Native American children^219^. This review was unable to identify any evidence that demonstrated treatment is as effective for indigenous women as it has been demonstrated for non-indigenous women^23^,^24^ or that demonstrated there are no detrimental psychological 54, social or physical consequences following GDM diagnosis, in either early or late pregnancy. Rather, one excluded abstract suggests this may be a greater concern for indigenous women 57.

Although this review highlights that the evidence-base is not sufficient to address the population-based screening criteria for indigenous women, studies in this review also demonstrate that more descriptive research alone is unlikely to improve health outcomes for indigenous women. Despite over 40 years of research in Pima Indian communities, which generated a predominance of high quality research compared with work from other indigenous communities 128, there has been little or no apparent improvement in related health outcomes.

There is an urgent need for strong evidence that demonstrates effective interventions for primary (prevention), secondary (early detection) and tertiary (treatment) prevention to mitigate the significant public health impact of DIP among indigenous women. All research in relation to DIP needs to consider equity. The level of uncertainty in the current evidence-base for population-based screening must be considered when introducing changes, and strategies should be employed to reduce the risks of intervening without sufficient evidence. These strategies include active collaboration and formative research with the communities involved and designing implementation plans with a capacity for reflective cycles and flexibility to respond to unforeseen consequences (e.g. action research), as well as comprehensive evaluation, so that learning can be shared with other communities. There is a need for evidence to develop strategies to improve consistency of screening during and after pregnancy. This should be informed by an understanding of women's preferences and values in relation to screening, evaluation of strategies where screening rates are high or low, and evidence from other screening programmes.

## Conclusion

Diabetes in pregnancy imposes an inequitable disease burden on indigenous women and their infants. Recent International Association of Diabetes and Pregnancy Study Groups recommendations to provide early pregnancy screening for GDM for women in populations with a high risk of T2DM^10^,^25^ offer potential benefits through earlier detection and offering an opportunity to provide effective interventions to reduce the risk for both the mother and her infant in the short and longer term 11. However, evidence is urgently needed to demonstrate that these potential benefits outweigh the risks, including that the early GDM screening recommendations are acceptable to indigenous women, and that once diagnosed, effective treatment and follow-up after pregnancy are available. Researchers, clinicians and policy makers must work together with communities to develop effective primary, secondary and tertiary strategies to reduce the impact of DIP in indigenous populations.

## Appendix A

### Sample search from Embase

1. exp "PARAMETERS CONCERNING THE FETUS, NEWBORN AND PREGNANCY" / or exp PREGNANCY/ or exp PREGNANCY OUTCOME/ or exp PREGNANCY COMPLICATION/
2. pregnan*.mp. [mp = title, abstract, subject headings, heading word, drug trade name, original title, device manufacturer, drug manufacturer, device trade name, keyword]
3. exp prenatal diagnosis/ or exp prenatal care/
4. antenatal.mp. [mp = title, abstract, subject headings, heading word, drug trade name, original title, device manufacturer, drug manufacturer, device trade name, keyword]
5. prenatal.mp. [mp = title, abstract, subject headings, heading word, drug trade name, original title, device manufacturer, drug manufacturer, device trade name, keyword]
6. exp PRENATAL GROWTH/ or exp PRENATAL STRESS/ or exp PRENATAL DISORDER/ or exp PRENATAL PERIOD/ or exp PRENATAL DIAGNOSIS/ or exp PRENATAL MORTALITY/ or exp PRENATAL DEVELOPMENT/ or exp PRENATAL CARE/ or exp PRENATAL SCREENING/ or exp PRENATAL EXPOSURE/
7. 1 or 2 or 3 or 4 or 5 or 6
8. newborn/
9. newborn*.mp. [mp = title, abstract, subject headings, heading word, drug trade name, original title, device manufacturer, drug manufacturer, device trade name, keyword]
10. neonatal.mp. [mp = title, abstract, subject headings, heading word, drug trade name, original title, device manufacturer, drug manufacturer, device trade name, keyword]
11. infan*.mp.
12. fetal.mp. [mp = title, abstract, subject headings, heading word, drug trade name, original title, device manufacturer, drug manufacturer, device trade name, keyword]
13. exp " EMBRYONIC AND FETAL FUNCTIONS" / or exp FETAL WELL BEING/
14. fetus.mp. [mp = title, abstract, subject headings, heading word, drug trade name, original title, device manufacturer, drug manufacturer, device trade name, keyword]
15. fetus/
16. foetal.mp. [mp = title, abstract, subject headings, heading word, drug trade name, original title, device manufacturer, drug manufacturer, device trade name, keyword]
17. foetus.mp. [mp = title, abstract, subject headings, heading word, drug trade name, original title, device manufacturer, drug manufacturer, device trade name, keyword]
18. fetus/
19. 8 or 9 or 10 or 11 or 12 or 13 or 14 or 15 or 16 or 17 or 18
20. 7 or 19
21. exp diabetes mellitus/
22. diabet*.mp. [mp = title, abstract, subject headings, heading word, drug trade name, original title, device manufacturer, drug manufacturer, device trade name, keyword]
23. 21 or 22
24. hyperglyc?mi*.mp. [mp = title, abstract, subject headings, heading word, drug trade name, original title, device manufacturer, drug manufacturer, device trade name, keyword]
25. exp hyperglycemia/
26. exp OBESITY/
27. obes*.mp. [mp = title, abstract, subject headings, heading word, drug trade name, original title, device manufacturer, drug manufacturer, device trade name, keyword]
28. exp glucose intolerance/
29. glucose intoleran*.mp. [mp = title, abstract, subject headings, heading word, drug trade name, original title, device manufacturer, drug manufacturer, device trade name, keyword]
30. 24 or 25 or 26 or 27 or 28 or 29
31. 23 or 30
32. 20 and 31
33. exp pregnancy diabetes mellitus/
34. gestational diabet*.mp. [mp = title, abstract, subject headings, heading word, drug trade name, original title, device manufacturer, drug manufacturer, device trade name, keyword]
35. 33 or 34
36. exp INDIGENOUS PEOPLE/
37. indigen*.mp. [mp = title, abstract, subject headings, heading word, drug trade name, original title, device manufacturer, drug manufacturer, device trade name, keyword]
38. exp ABORIGINE/
39. aborigin*.mp. [mp = title, abstract, subject headings, heading word, drug trade name, original title, device manufacturer, drug manufacturer, device trade name, keyword]
40. 36 or 37 or 38 or 39
41. 32 or 35
42. 40 and 41
43. 31 and 40
44. 20 and 40
45. limit 42 to yr = “2010 -Current”
46. torres strait*.mp. [mp = title, abstract, subject headings, heading word, drug trade name, original title, device manufacturer, drug manufacturer, device trade name, keyword]
47. first nation*.mp. [mp = title, abstract, subject headings, heading word, drug trade name, original title, device manufacturer, drug manufacturer, device trade name, keyword]
48. American Indian/
49. native*.mp.
50. Eskimo/
51. alaska* native*.mp. [mp = title, abstract, subject headings, heading word, drug trade name, original title, device manufacturer, drug manufacturer, device trade name, keyword]
52. Maori/
53. maori*.mp. [mp = title, abstract, subject headings, heading word, drug trade name, original title, device manufacturer, drug manufacturer, device trade name, keyword]
54. inuit*.mp.
55. 46 or 47 or 48 or 49 or 50 or 51 or 52 or 53 or 54
56. (41 and 55) not 42
57. *American Samoa/
58. american samoa*.mp. [mp = title, abstract, subject headings, heading word, drug trade name, original title, device manufacturer, drug manufacturer, device trade name, keyword]
59. native hawai*.mp. [mp = title, abstract, subject headings, heading word, drug trade name, original title, device manufacturer, drug manufacturer, device trade name, keyword]
60. aleut.mp.
61. 57 or 58 or 59 or 60
62. (61 and 41) not (42 or 56)
