# Supplementary material for: Diabetes in pregnancy among indigenous women in Australia, Canada, New Zealand and the United States: a systematic review of the evidence for screening in early pregnancy
Source: Diabetes Metab Res Rev. 2013 May 5;29(4):241–56. doi: 10.1002/dmrr.2389 (PMC3698691; doi:10.1002/dmrr.2389)
Supplement: Supplementary file 1 [file dmrr0029-0241-SD1.docx]

# Diabetes in Pregnancy among Indigenous Women in Australia, Canada, New Zealand and the United States:

# A systematic review of the evidence for screening in early pregnancy -Characteristics of Included Studies

| **Study ID (First author/publication year; study duration if specified)** | **Population (Country; Indigenous community; urban/rural/remote/mixed/or unknown; population/community or clinic based data)** | **Publication details (Intervention, descriptive, measurement or other; Study design; Hypothesis; number included; data source; diagnostic criteria used if applicable)** | **Main *theme* and outcomes** | **Risk of bias appraisal (mod-high risks specified) and identified funding source.** |
| --- | --- | --- | --- | --- |
| Adams 1970 (study 1967-68) | United States (Arizona); Papago Indian; Remote Reservation; Community based data | Descriptive (quantitative); prospective cohort; Study to examine effect of maternal DIP on birthweight; n=124 infants & 900 school-aged children; hospital record of BSL >150mmol after 75g OGTT & direct measurement for T2DM at 1 year. | *Epidemiology and Natural History/risk factors for baby during pregnancy and birth:* Increased birthweight in mothers with DIP (average 128 ounces) than without DIP (average 117.6 ounces). | Low risk of bias |
| Alberti 2004 | International | Consensus meeting of T2DM in offspring | *Epidemiology and natural history/long term risk factors for baby*: Maternal DIP increased risk of T2DM to offspring | NA: expert consensus workshop |
| Aljohani 2008 (1985-2004) | Canada (Manitoba); First Nations; mixed; population based data | Descriptive (quantitative); retrospective cohort; study to examine impact of GDM on birth outcomes; n=324605 births; Prenatal Health database; After 1992 used SCOG criteria ("abnormal FPG using CDA guidelines" after 50g GCT and 75-100g OGTT). | *Epidemiology and natural history/risk factors for mother and offspring:* FN status in combination with GDM increased risk of shoulder dystocia. | Moderate risk of outcome assessment bias as relies on birth registry database. Funded by Canadian Institute of Health Research. |
| Aljohani 2008b (1985-2004) | Canada (Manitoba); First Nations; mixed; population based data | Descriptive (quantitative); retrospective cohort; study to examine impact of GDM on birth outcomes; n=324605 births; Prenatal Health database; After 1992 used SCOG criteria ("abnormal FPG using CDA guidelines" after 50g GCT and 75-100g OGTT). | *Epidemiology and Natural History/ Prevalence DIP:* GDM 6.9% (2.6-3.8 times higher in FN than non-FN women during 1985-2004), Higher prevalence in rural (7.3%) than urban (5.8%) areas. | Moderate risk of outcome assessment bias as measurement from birth registry database. Funded by Canadian Institute of Health Research. |
| Alur 2002 (2000-2002) | United States (Guam); Chammorro; Unknown if urban/remote; clinic based data | Descriptive (quantitative); retorspective cohort to investigate DIP prevalence; Obstetric records reviewed; diagnostic criteria 1 hr 50g OGTT | *Epidemiology and natural history: prevalence:* Prevalence DIP 5.5% (crude rate), which is significantly more than non-Chammorro population | High ROB (selection bias unclear; outcome assessment from medical records; limited analysis). Funding source not specified. |
| Anonymous 2000 | Canada (James Bay); Cree; | Other (commentary from Indiegnous perspective) | *Other: preventive interventions:* Recommends interventions have greater community involvement | NA:commentary |
| Australian Institute of Health and Welfare 2008 (2002-2007) | Australia; Aboriginal and Torres Strait Islander; mixed; population based data | Descriptive (quantitative) cross-sectional study of GDM prevalence; n=3469 women; survey of general practitioners; diagnostic criteria unclear | *Epidemiology and natural history/ prevalence:* Increased prevalence of GDM (7.8% crude; 10.9% age adjusted) | Moderate risk of bias from outcome assessment via doctor survey. Funded by Australian Government. |
| Benjamin 1993 (1989-1991) | United States (New Mexico); Zuni Indian; remote reservation; clinic based data | Descriptive (quantitative); retrospective cohort/case control study to determine prevalence DIP nad progression to T2DM; n=809 (CCS n=51); maternity service records & birth registry; diagnostic criteria 1hr 50g OGTT at 24-28 weeks and 100g 3hr OGTT if over 7.8mmol (O'Sullivan criteria) | *Epidemiology and natural history: prevalence and risk to birth outcomes:* GDM 14.5 % crude and DIP 15.3% crude with 30% progressing to T2DM after a mean of 4.8 years (followup up to 9 years but average only 5.5 years). | Moderate risk of outcome bias as medical record diagnosis and analytical bias as no confidence intervals reported. Funding source not specified. |
| Bennett 1976 (1966) | United States (Arizona); Pima Indian; remote reservation; community based study data | Descriptive (quantitative); propsective cohort to determine prevalence of DIP and outcomes; n=1253 pregnancies (82% of community); Medical records; diagnostic criteria 2hr 75g OGTT =>160mmol (WHO). | *Epidemiology and natural history: prevalence and risk to birth outcomes:* High prevalence of DIP 3.8% (10-15 times higher than general population) and poor outcomes amongst diabetic pregnancies. | Moderate risk of analytical bias as limited adjustment for confounders. Funding source not specified. |
| Bennett 1978 (1965-1977) | United States (Arizona); Pima Indian; remote reservation; community based study data | Descriptive (quantitative); propsective cohort to determine rate of congenital abnormalities amongst diabetic and non-diabetic mothers; n=2648 offspring; Medical records; diagnostic criteria 2hr 75g OGTT =>160mmol (WHO). | Epidemiology and natural history/risk to baby suring pregnancy and birth: DIP 4.3% crude rate, and congenital anomalies 4 times higher in diabetic pregnancies (11.4%). No effect if father diabetic. | Low risk of bias. Funding source not specified. |
| Benyshek 2001 | United States; Native American; | Other (review). | *Epidemiology and natural history/long term risk to baby:* Describes fetal origins of T2DM stimulated by malnourished "thrifty phenotype" with low insulin resistance which started generational cycle. | Literature review (no search criteria or quality appraisal described). Funding source not specified |
| Bhattarai 2009 | International | Other (review). | *Epidemiology and natural history/long term risk to baby*: Argues that DIP is a major factor contributing to the high rates of T2DM in Indiegnous communities | Literature review (no clear question, search criteria or quality appraisal described). Funding source not specified |
| Blair 1994 (1985-1987) | Australia (West Australia); Aboriginal; mixed; clinic based data | Descriptive (quantitative); retrospective cohort to determine effect of gestational age on bw; n=1301; midwives perinatal data collection; diagnostic criteria is diagnosis in medical records only | *Epidemiology and natural history/risks to baby during pregnancy and birth;* 19/24 infants with unusually high birthweight all had DIP | Moderate risk of outcome assessment bias from medical records. Funding source not specified. |
| Blair 1996 (1985-1987) | Australia (West Australia); Aboriginal; mixed; clinic based data | Descriptive (quantitative); retrospective cohort to review factors associated with low birth weight; n=1301; midwives perinatal data collection; diagnostic criteria is diagnosis in medical records only | Epidemiology and natural history/risks to baby during pregnancy and birth; DIP 12.2% (crude). DIP was one pathological factor accounting for low birth weight. | Moderate risk of outcome assessment bias from medical records. Funding source not specified. |
| Bogardus 2002 (1965) | United States (Arizona); Pima Indian; remote reservation; community based study data | Descriptive (quantitative); prospective cohort/longitudinal study to examine if insulin secretion is associated with development of T2DM; n=300; Medical records and direct examination; diagnostic criteria not specified; 7 year followup. | *Epidemiology and natural history/long term risk to baby:* DIP is associated with a reduced Acute Insulin Response in offspring. | Low risk of bias. Funding source not specified. |
| Bower 1992 (1980-1984) | Australia (West Australia); Aboriginal; mixed; population based data | Descriptive (quantitative); retrospective to determine prevalence of birth defects amongst women with DIP; n=11019; midwives data collection records; diagnostic criteria not specified (diagnosis recorded in database) | *Epidemiology and natural history/risk to baby during prengnancy and birth:* Prevalence GDM 1.33% (crude), T2DM 0.35% (crude) and DIP 1.79% (crude). Increased risk of birth defects amongst women with DIP. | Moderate risk of outcome assessment bias as diagnosis relies on medical record in database. Funded by AMP society. |
| Brennand 2005 (1994-2000) | Canada (James Bay, Quebec); Cree; remote; community based data | Descriptive (quantitative); retrospective cohort to investigate association between pregnancy weight gain and outcomes; n=603; Medical records; diagnostic criteria not specified (antenatal diagnosis in records). | *Epidemiology and natural history/risk to baby during pregnancy and birth:* GDM 18.6% crude (aggregated by weight), DIP 27.4% crude (only 23% had normal weight). Excessive weight gain and pre-existing obesity are associated with poor outcomes, regardless of DIP. | Moderate selection bias as 85% missing data and high proportion of overweight women, and outcome assessmwent bias as based on medical records. Funded by Cree Board of Health and Social Services. |
| Bunt 2005 (2001-2003) | United States (Arizona); Pima Indian; remote reservation; community based study data | Descriptive (quantitative); retrospective cohort investigating association of maternal DIP and T2DM in offspring; n=42; medical records; diagnostic criteria 75g 2hr OGTT >11.1 mmol/L (WHO). | *Epidemiology and natural history/long term risks to baby:* Maternal DIP increases HbA1c in offspring (children 7-11years) | Low risk of bias. Funded by National Institute of Health. |
| Campbell 2002 | Canada; Cree | Other (Discussion) | *Other/preventive interventions:* Authors argue that T2DM is a result of marginalisation and recommends structural improvement in health care rights and recognition of cultural contexts. | NA: discussion only. Funding source not specified. |
| Campbell 2012 (1998-2008) | Australia (Far North Queensland); Aboriginal and Torres Strait Islander; rural&remote; community based data. | Descriptive (quantitative); prospective cohort to investigate associations of pre-pregnancy health and DIP; n=220; linked survey and hospital & perinatal data; diagnosis in medical records. | *Epidemiology and natural history (Prevalence and risk for maternal development of DIP):* DIP prevalence 10.4%. Women who developed DIP were on average 5 years older, had higher BMI's, and elevated pre-pregnancy fasting BGL. | High risk of selection bias (44.5% self-selected sample) and moderate risk of outcome assessment bias as relies on database diagnosis of DIP. |
| Caughey 2010 (1995-1999) | United States; Native American; mixed; population based data. | Descriptive (quantitative); retrospective cohort to investigate association of maternal and paternal race with GDM; n=800 Native American mothers; Linked hospital data; 50g OGCT (database diagnosis). | *Epidemiology and natural history (Prevalence):* Maternal GDM prevalence second highest rate among Native American mothers (5.6%) and 4.5% had Native American partners. | Moderate risk of outcome assessment bias as relies on database for diagnosis. |
| Caulfield 1998 (1990-1993) | Canada (Ontario); Cree; mixed population; clinic based data | Descriptive (quantitative); retrospective cohort to investigate factors associated with macrosomia; n=1652 singleton births; medical records; diagnostic criteria 50g 1h OGCT at 24-28 weeks then 100g OGTT (90%) (O'Sullivan criteria). | *Epidemiology and natural history/ risk to baby during pregnancy and birth:* T2DM associated with highest risk of macrsomia, followed by GDM. | Moderate risk of outcome assessment bias as relies on medical records. Funding source not specified. |
| Charles 1994 (1965-1991) | Unites States (Arizona, Gila River community); Pima Indian; remote; community based data. | Descriptive (quantitative); longitudinal study to investigate pathogenesis of hypertension; n=1698 children; direct source data based on 2h 75g OGTT. | *Epidemiology and natural history (Long term risks to infant):* Association between maternal, but not paternal, diabetes and high blood pressure among children. | High risk of selection bias as participation rate not reported. |
| Claydon 2007 (2002-2004) | Canada;Aboriginal; mixed; population based data | Descriptive (quantitative); prospective cohort to investigate factors associated with race in infants admitted to intensive care; n=9502 infants; prospectively collated database for 2 years. | Epidemiology and natural history/risks to baby during pregnancy and birth: Aboriginal mothers had more perinatal risk factors and higher rates of macrosomia, and authors suggest may be different racial manifestations of risk. | Moderate risk of outcome assessment bias as dependant on medical records. Funded by the Canadian Institute of Health Research. |
| Cleary 2006 (1999-2003) | Canada (Manitoba); First Nations; | Intervention; before after evaluation of education intervention to improve screening for GDM; n=64; Medical records; diagnostic criteria 1 hr 50g OGTT at 24-28 weeks gestation. | *Screening practice and rates*: Education improved screenign practice but it remained suboptimal. Recommend all care providers included in education. | High risk of bias as not randomised. Sourc eof funding not specified. |
| Cloutier 2009 (2008) | Canada (Manitoba); First Nations; population unknown; community based data | Descriptive (quantitative); retrospective cohort to measure ht and wt of offspring of mothers with T2DM; n=40 children; medical examination; T2DM diagnosed prior to pregnancy | *Epidemiology and natural history/long term risk to baby:* 100% obesity in boys and 68% in girls. 44% of children over 10 years had T2DM. Two were diagnosed before 10 years of age. | Selection bias and analytical bias unclear as only conference abstract available. Funding source not specified. |
| Comess 1969 (1965-1967) | United States (Arizona); Pima Indian; remote reservation; community based study data | Descriptive (quanitative); retrospective audit of medical records to investigate rate of congenital abnormalities according to maternal diabetic status; n=237 women and 1207 children; medical records; diagnostic criteria 2h 75g OGTT >160 mg. | *Epidemiology and natural history/ risks to baby during pregnancy and birth:* Increased risk of congenital abnormalities amongst offspring of mothers with DIP, but not fathers. Risk highest amongst offspring of mothers requiring medication. | Moderate risk of outcome assessment bias and analytical bias as limited adjustment for confounders. Funding source not specified. |
| Congress Alukra 1999 | Australia (Northern Territory); Aboriginal; | Other (guideline) | *Adequate treatment pathways:*Recommends early screening and management | NA: guideline only. |
| Conroy 2006 | Canada; Aboriginal | Other (review) | *Other/prevention:* 3/6 childhood obesity prevention programs in Canada addressed GDM amongst Aboriginal women. | Comprehensive search strategy and inclusion criteria but no risk of bias appraisal. No funding sourced specified. |
| Coory 2000 (1988-1997) | Australia (Queensland); Torres Strait Islander; mixed; population | Descriptive (quantitaive); retrospective cohort to investigate if bw is an appropriate outcome measure for TSI babies; n=5000 live births; Perinatal data collection by midwives; antenatal diagnosis on data collection form; | *Epidemiology and natural history/risks to baby during pregnancy and birth*: Despite higher bw for TSI babies, mortality still as high as Aboriiginal babies. | Moderate risk of bias as relies on diagnosis on medical data collection form; Source of funding not specified. |
| Cundy 1993 (1987-1989) | New Zealand; Maori; mixed; clinic based data. | Descriptive (quantitative); retrospective cohort to examine effects of ethnicity, glycaemic control, adiposity and smoking on birthweight; n=220; clinic and hospital data; Direct diagnosis using 1h 50g OGCT >7.8mmol/l and 3h 100g OGTT >50mmol/h. | *Epidemiology and natural history (risk to infant in pregnancy and birth):* DIP associated with increased birthweight but no difference between GDM and NIDDM, despite poorer glycaemic control. Positive association between maternal BMI and pregnancy weight gain and infant birthweight, and negative association between maternal BP and smoking and infant birthweight. | High risk selection bias as unclear how controls selected. |
| Dabelea 1998 (1967-1996) | United States (Arizona); Pima Indian; remote reservation; community based study data | Descriptive (quantitative); retrospective cohort investigating the risk factors associated with T2DM in children; n=5274 children aged 5-10; medical examination; 1985 WHO diagnostic criteria | *Epidemiology and natural history/long term risk to baby:* T2DM in children associated with increased weight and exposure to DIP | Low risk of bias. Funding source not specified. |
| Dabelea 2000 (1967-1996) | United States (Arizona); Pima Indian; remote reservation; community based study data | Descriptive (quantitative); retrospective cohort investigating the risk factors associated with T2DM in children; n=125 children aged 5-10 with T2DM; medical examination; 1985 WHO diagnostic criteria | *Epidemiology and natural history/long term risk to baby:* Exposure to T2DM accounted for all cases of T2DM under 15 years and 40% under 19 years. | Low risk of bias. Funding source not specified. |
| Dagogo-Jack 2010 | Indigenous/ethnic. | Review of use of HBA1C among different population groups. | *Screening test efficacy and cost:* HBA1C useful for monitoring diabetes and predicting microvascular complications but not as a diagnostic tool as significant variability due to biological and genetic factors. | Not a systematic review: no specific question, search strategy or quality appraisal described. |
| Dannenbaum 1999 | Canada (James Bay); Cree. | Intervention (Program description) | *Systems for followup*: description of development of a register to followup women diagnosed with GDM. | NA: program description only. Funding source not specified. |
| Davis 2009 (2001-4) | Australia (North Queensland); Aboriginal and Torres Strait Islander; mixed population; uses population based data | Descriptive (quantitative); retrospective audit to investigate perinatal outcomes associated with DIP; n=2044 women; medical records linked to databases; diagnostic criteria 2h 75g OGTT >9mmol | *Epidemiology and natural history/prevalence and risks to mother and baby:* Prevalence DIP 6.7% crude, and increased risk of caesarean, hypoglycaemia, respiratory distress, less normal birthweights and full terms deliveries associated with DIP. | Moderate risk of outcome assessment bias but linked records. Funding source not specified. |
| Denny 2012 (2008) | United States; Native American/Alaska Native; mixed; population based data. | Descriptive (quantitative); cross sectional survey to examine pre-conceptual risk factors among women 18-44 years; n=1127/54,612; survey data; self-reported diagnosis. | *Epidemiology and natural history (prevalence):* Prevalence diabetes highest among AI/AN non-pregnant women of childbearing age (10%). AI/AN women also had highest rate of 2 or more pre-conceptual risk factors (34.4%). | High risk of outcome assessment bias as self-reported diabetes. |
| Devlin 2008 (1993-2003) | United States: American Indian; mixed; population based data. | Descriptive (quantitative); retrospective cohort to examine trends in DIP among population groups; n=2035 AI women; birth record data; diagnosis on database. | *Epidemiology and natural history (prevalence/trends):* Non-significant increase from 3.98% (1993) to 4.6% (2003). Highest rates at baseline, but increases higher among other population groups (particularly Asian women) so disparities decreasing. | Moderate risk of outcome assessment bias as relies on recording of diagnosis in database. |
| Dooley 1998 (1989-1992) | Canada (Sioux lookout zone); Ojibwa-Cree; remote; clinic based data | Descriptive (quantitative); retrospective audit to investigate pregnancy outcomes of T2DM in pregnancy; n=26 children; medical records; medical diagnosis | *Epidemiology and natural history/risks to baby during pregnancy and birth:* Describes births outcomes of DIP | High risk of bias as no control group, limited analysis, reliant on medical record diagnosis. Source of funding not specified. |
| Doran 2007 | Australia (New South Wales); Aboriginal; urban | Descriptive (qualitative); focus group with women to discuss attitudes to exercise in pregnancy; n=4; transcripts | *Other/prevention:* Limited understanding of how to incorporate exercise into pregnancy. | Potential risk of bias as research question not clear; researcher bias not explicit and conclusions may not be justified by results. Funding source not specified. |
| Dyck 1998 (1994-5) | Canada (Saskatoon); Aboriginal; remote | Intervention; pilot study to determine if project to promote exercise in pregnancy is feasible; n=5; diagnostic criteria 3h 100g OGTT | *Other/prevention:* Challenges to recruitment, which improved with incentives. | High risk of bias as no control group (description only). No source of funding identified. |
| Dyck 2001 (1991-1994) | Canada (Saskatoon); Saskatchewan Indian; mixed; population based data | Descriptive (quantitative); case-control study to investigate the association between high birthweight and T2DM; n=1366x4; ICD-code data | *Epidemiology and natural history/long terms risk to baby:* Significant association between HBW and T2DM. | Moderate risk of outcome assessment bias as diagnosis relies on ICD dataset. Source of funding not specified. |
| Dyck 2002 (1998) | Canada (Saskatoon); Saskatchewan Indian, Metis and Inuit; mixed; clinic based data | Descriptive (quantitative); prospective cohort to determine the prevalence, risk factors and outcomes of GDM; n=2006 women; questionaire, logbook and medical records; range of diagnostic criteria accepted | *Epidemiology and natural history/prevalence:* GDM 11.5% for Aboriginal women and 3.5% for non-Aboriginal women. Aboriginality an independent risk factor, particularly when combined with obesity. | Low risk of bias. Thdere was 26% attrition but the differences were accounted for in analysis. Funded by Saskatoon Health District. |
| Dyck 2005 | Canada (Saskatoon); Saskatchewan Indian, Metis and Inuit; | Other (Review) | *Epidemiology and natural history/long term risk to baby:* DIP plays a key role in perpetuating the T2DM epidemic in Aboriginal communities. | Literature review of authors own studies and selected other studies. No clear search question, search strategy or risk of bias appraisal. Source of funding not specified. |
| Dyck 2010 (1980-2005) | Canada (Saskatoon); First Nations; mixed; population based data | Descriptive (quantitative); retrospective cohort to invesitgate the epidemiology of T2DM; n=8275 FN & 82306 non-FN; MoH insurance registry data; diagnostic criteria based on hospital separation codes, medical claims or ICD codes. | *Epidemiology and natural history/prevalence:* Prevalence T2DM 4x higher in FN women and occurs at an earlier age, disproportionately affecting women of reproductive age. Prevalence in women increased from 9.5% to 20.3% over study period. | Moderate risk of outcome assessment bias as relies on database. No funding source specified. |
| Dyck 2010 (1984-2001) | Canada (Saskatchewan); First Nations; mixed; population based data. | Descriptive (quantitative);case control study to examine association of birth-related factors and diabetes risk in offspring; n=1366 FN people with diabetes and 1366 other Saskatchewan's; linked birth record and health data; diagnosis on database. | *Epidemiology and natural history (risk to infant in pregnancy and birth/and long term risk to infant):* Increased maternal age and parity were associated with an increased risk of diabetes. DIP was associated with increased risk of infant macrosomia. | Moderate risk of outcome assessment bias as relies on recording of diagnosis in database. |
| Fajans 1993 | United States; Native American; | Other (workshop report) | *Adequate treatment pathways:* Asks if current definitions of GDM optimal for Native Americans | NA:discussion only |
| Falhammar 2010 (1999-2006) | Australia (North Queensland); Torres Strait Islander; remote population; uses clinic based data | Descriptive (quantitative); retrospective cohort to audit births from two time periods to compare screening practice and prevalence; n=454; medical records; diagnostic criteria RBGL at first visit, 24, 28 and 34 weeks, with 50g OGCT if over 5.5 mmol and 75g OGCT if over 7.8mmol. | *Epidemiology and natural history + screening practice and rates:* Prevalence GDM 3.5% (1999) & 8.7% (2006) crude rates. DIP 4.3% (1999) & 13.3% (2006) = 4-fold increase. 99.5% screened but identified need to improve followup. | Moderate risk of selection bias as 18% delivered at other hospitals and outcome asssessment bias as relies on medical record data. Funding source not specified. |
| Franks 2006 (1965-2004) | United States (Arizona); Pima Indian; remote reservation; community based study data | Descriptive (quantitative); prospective longitudinal study to investigate association between pregnancy gluocse levels and T2DM in children; n=911 women & 1436 children; medical examination; diagnostic criteria 2h 75g OGTT >11.1mmol; up to 39 year followup | *Epidemiology and natural history/long term risks to baby:* Maternal glycaemia associated with higher birthweight and T2DM in offspring even when mothers are normal glucose tolerant in pregnancy | Low risk of bias. Funded by National Institute of Diabetes and Digestive and Kidney Diseases intramural program. |
| Franks 2007 (unclear data collection dates) | United States (Arizona, Gila River); remote; community based data. | Descriptive (quantitative); longitudinal study to identify early life risk factors for T2DM in children; n=1604; study data; 2h 75g OGTT WHO diagnostic criteria. | *Epidemiology and natural history(long term risk to infant):* Exposure to intrauterine diabetes increases risk of T2DM in offspring. | High risk of selection bias as 32.6% longitudinal study participants did not return for follow-up. |
| Gaudreau 2012 (2006) | Canada (Quebec); 2 Alonquin communities; remote; community based data. | Descriptive (qualitative); interviews with women with GDM to identify cultural factors which maintain health behaviours; n=15. | *Preferences or values:* Helpful themes identified include: Importance of family and social ties, possibility of preserving cultural values, opportunity to learn behaviours through resources which are culturally adapted to needs, the chance of saving money through better diet and access to blood sugar data. | Research by non-Indigenous researchers but results approved and modified by informants. Moderate quality research. |
| Gautier 2001 (1965-NS) | United States (Arizona); Pima Indian; remote reservation; community based study data | Descriptive (quantitative); prospective longitudinal study to determine if insulin secretion is affected in offspring of adult with early onset T2DM; n=104;medical examination; diagnostic criteria 2h 75g OGTT . | *Epidemiology and natural history/ long term risks to baby:* Lower Acute Insulin Response in offspring of diabetic mothers | Low risk of bias. Funded by Diabetes Research and Training Centre. |
| Godwin 1999 (1987-1995) | Canada (James Bay); Cree; mixed population; clinic based data | Descriptive (quantitative); retrospective cohort to determine prevalence of GDM; n=1298 women; diagnostic criteria FPG or 1h 50g OGTT .7.8mmol or O'Sullivan criteria (Metzer 1991) | *Epidemiology and natural history/prevalence & risks to mother:* Prevalence GDM 8.5% crude. Increased risk with age. GDM associated with increased risk of caesarean. | Moderate risk of outcome assessment bias as uses medical record data. Funding source not specified. |
| Gohdes 2004 (1989-2000) | United States (Montana); Mixed Indian tribes; | Other (review) of reports of prevalence. | *Epidemiology and natural history/prevalence:* Prevalence DIP 3.1% (1989-91) to 4.1% (1998-2000) crude rate. | Literature review. No clear question, search strategy or quality appraisal of included studies. Funding souorce not specified. |
| Gray-McDonald 2000 (1995-1997) | Canada (James Bay, Quebec); Cree; remote; | Intervention; controlled trial to evaluate dietary counselling and exercise; n=219 | *Other/prevention:*No significant effect from diet and exercise intervention. | High risk of bias as not randomised. But did have a control group. 87% participation. Funding source not specified. |
| Hadden 1985 | International | Other (review); Prevalence of GDM | *Epidemiology and natural history/prevalence:* Prevalence GDM amongst Pima Indian women the highest in the world (25/1000/year). | Literature review. No clear question, search strategy or quality appraisal reported. Funding source not identified. |
| Hamilton 2004 | Canada (Nunavut Arctic); Inuit; remote | Intervention; Process evaluation of online nutrition education and booklet for health care workers; n=96. | *Other/prevention:* 40% participation in course and program described in detail. | High risk of bias as not randomised and process evaluation only. Funding source not specified. |
| Harris 1997 (1990-1993) | Canada (Ontario); Ojibwa-Cree; mixed population; clinic based data | Descriptive (quantitative); Retrospective audit to determine prevalence of DIP; n=1305 births; Hospital records; diagnostic criteria 50g OGTT at 24-28 weeks then 1h 100g OGTT if over 7.8mmol. | *Epidemiology and natural history/prevlaence:* Prevalence DIP 11.6% (crude) and GDM 8.4% (crude). Risk increased with age, doubling every 5 years. | Moderate risk of bias from outcome assessment. Fuding source not specified. |
| Hart 1985 (1981-82) | Australia (South Australia); Aboriginal; mixed; population based data | Descriptive (quantitative); Retrospective audit to describe risk factors in birth; n=550 mothers + 555 infants; midwives data collection; diagnostic criteria based on diagnosis recorded on form | *Epidemiology and natural history/prevalence and risks to mother and baby:* Prevalence 2% (crude rate) and increased risk factors amongst Aboriginal women (including DIP). | Moderate risk of bias from outcome assessment reliant on midwives data collection and high risk of bias as no adjustment or analysis for confounding factors. Source of funding not specified. |
| Hughes 2006 (1998-2002) | New Zealand (Auckland); Maori and Pacific Islander women combined; mixed; clinic based data | Descriptive (quantitative); retrospective cohort to determine pregnancy outcomes of women with T2DM; n=214; medical records; diagnostic criteria based on medical diagnosis | *Epidemiology and natural history/prevalence & risks to mother and baby:* Reported T2DM in pregnancy 8.4% (crude). Significant difference in T2DM and birth outcomes (but confidence intervals overlap) | Moderate risk of bias from outcome assessment dependant on medical records. Funding source not specified. |
| Humphrey 2000 | Australia (North Queensland); Aboriginal and Torres Strait Islander; remote population; clinic based data | Descriptive (quantitative); retrospective cohort of prevalence; See Kim 1999 for details | *Epidemiology and natural history/prevalence:* GDM19.7% & 21.1% (1992); 7% &14.4% (1996). Significant decrease in GDM 1992-1996. | See Kim 1999. |
| Hunt 2007 | Australia; Aboriginal and Torres Strait Islander; | Other: GDM management advice | *Adequate treatment pathways:*Recommends early GDM screening | NA: book chapter. |
| Hunt 2007b | International | Other: Review of prevalence of DIP | *Epidemiology and natural history/prevalence & screening practice:* Prevalence increasing and describes changes in diagnostic criteria over time | Clear question, seach strategy, risk of bias appraisal of inluded studies, including differentiation between different types of data. Funding source not specified. |
| Ishak 2003 (1988-1999) | Australia (South Australia); Aboriginal; mixed; population based data | Descriptive (quantitative); retrospective cohort to investigate GDM incidence and trends; n=230011 births; Perinatal data based on midwives data collection; diagnostic criteria based on medical diagnosis | *Epidemiology and natural history/prevalence:* Prevalence GDM 4.29% (crude), with the age-standardized rate 2.5 x higher than non-Aboriginal women; and T2DM in pregnancy 1.75%, which is 5 times higher than non-Aboriginal women. | Moderate risk of bias from outcome assessment dependant on midwives data collection. Funding source not specified. |
| Jaiyeola 2009 | United States; Native American; | Other: Review of causes of health disparities | *Epidemiology and natural history/long term risk to baby:* Discusses implications of high prevalence for next generations | NA: book chapter |
| Jin 2002 (1993-1997) | Canada (British Columbia); First Nations; mixed; population based data | Descriptive (quantitative); Retrospective cohort to describe rates of hospitalization associated with DM; n=1626; Hospital discharge data; Diagnostic criteria ICD9 codes. | *Epidemiology and natural history/risks to moether during pregnancy and birth:*Pregnant women of Indian status over 35 years old twice as likely to be hospitalized for DIP complications (no difference under 35 years) | High risk of outcome assessment bias from diagnosis based on ICD codes and moderate risk of bias from limited adjustment for confounders. Funding source not specified. |
| Johnson 2002 (1987-1997) | Canada (British Columbia); First Nations; mixed; population based data | Descriptive (quantitative); Cross-sectional survey to determine prevalence of DM; n=24407 (82 services); Survey data; Survey asks hosptial staff the number of women who had GDM. | *Epidemiology and natural history:* Prevalence GDM has doubled in 10 years. GDM 28/1000 live births (crude). | High risk selection bias as only 50/82 servicxes participated, and outcome assessment bias as relies on survey, limited adjustment or analysis for confounders. Funding source not specified. |
| Joshy 2006 | New Zealand; Maori | Other: Review of DM prevalence and screening practice | *Epidemiology and natural history & screening practice and rates:* High proportion undiagnosed T2DM as GDM in pregnancy for Maori women (21%) and high rates GDM (7.9%), but low rates screening (47.3%) | Clear review question, search strategy and inclusion criteria but risk of bias appraisal not described. Funding source not specified. |
| Kieffer 1999 (1994-1996) | United States; Native American; mixed; population based data | Descriptive (quantitative); retrospective cohort to investigate prevalence of DIP according to ethnicity and birthplace; n=10854224; birth certificate data; diabetes listed as a risk factor on birth certificate. | *Epidemiology and natural history/prevalence:* Prevalence DIP 4.44% crude, which is the second highest rate in US (Asian-Indian women had the highest rate). | High risk of outcome assessment bias as relies on birth certificate data. Funding source not specified. |
| Kim 1999 (1992-1996) | Australia (North Queensland); Aboriginal and Torres Strait Islander:remote; clinic | Descriptive (quantitative); retrospective cohort to investigate trends in GDM prevalence; n=7576 births; hospital database and medical records; diagnostic criteria based on medical records | *Epidemiology and natural history/prevalence:* GDM19.7% & 21.1% (1992); 7% &14.4% (1996). Significant decrease in GDM 1992-1996. | Moderate risk of outcome assessment bias which relies on medical records and limited adjustment for confounders. Funding source not specified. |
| Kim 2012 (2004-2007) | United States (Florida); American Indian; mixed; population based data. | Descriptive(qualitative); retrospective cohort to estimate race/ethnicity-specific percentage of GDM attributable to overweight and obesity; n=1252 AI women; birth registry data; diagnosis recorded in database. | *Epidemiology and natural history (prevalence and risk for maternal development of DIP):* DIP prevalence 6.5%, with 52.8% attributable to overweight and obesity. Asian Indian and Pacific Islander women had a higher prevalence (9.9%) but only 15.1% attributable to overweight and obesity. | Moderate risk of outcome assessment bias as diagnosis relies on reporting in database. |
| Klomp 2003 (1995-1997) | Canada (Saskatoon); Saskatchewan Indian; urban | Intervention: Description of an exercise program; n=35 women; evaluation based on field notes | *Other/prevention:* Challenges to attract and retain participation | Process evaluation only so high risk of bias. Funded by Health Canada NHRDP. |
| Kmetic 2008 | Canada; First Nations | Other: Review of prevalence and risk factors for CVD and T2DM using a "lifecourse perspective". | *Epidemiology and natural history/long term risks to baby:* High prevalence GDM and DIP, which heralds risk to mother and has an effect on offspring. Protective effect of breastfeeding. | Clear review question but search strategy, inclusion criteria and risk of bias appraisal not described. Funding source not specified. |
| Kuberski 1980 (1976) | United States (Guam); Chammorro; mixed; population based data | Descriptive (quantitative); retrospective cohort to assess impact of DM; 3048 live births; birth and death certificates; record of DM on certificates | *Epidemiology and natural history/prevalence and risk to mother:* DIP 0.75% (crude). Authors conclude most complications of DIP are likely to go unrecognised. | High risk of outcome assessment bias as diagnosis depends on birth certificate record and moderate-high risk of analytical bias as limited analysis and adjustment for confounding. Funding source not specified. |
| LaVaille 2003 (1989-1991) | United States; Native American and Alaskan Indian; mixed; population based data | Descriptive (quantitative); retrospective cohort to describe perinatal outcomes amongst NA/AI women with DIP; n=242715 births, 115187 NA/AI; National centre for health statistics database; diagnostic criteria is medical diagnosis on birth certificate | *Epidemiology and natural history/prevalence and risk to mother and baby:* Prevalence DIP 3.6% (crude) and NA/AI women had less prenatal care and higher rates of macrosomia. | High risk of outcome assessment bias as relies on birth certificate record and mod-high risk of analytical bias as no apparent adjustment for confounding. Funding source not specified. |
| Lavallee 2011 | International (Indigenous). | Commentary from Indigenous perspective. | *Preferences and Values:* Suggests researchers look to 'root cause' of risk factors and viewing them as symptoms of broader social determinants, rather than focussin on those alone. Suggests researchers engage with communities and advocate for social change drive by the community, as well as considering how the research helps or harms the community. | Commentary only, but from an Indigenous perspective. |
| Lindsay 2000 (1955-1994) | United States (Arizona); Pima and Tohono Indian; remote; community based data | Descriptive (quantitative); retrospective analysis (of longitudinal study) to investrigate trends in birthweight, BMI and DM in offspring of DIP; n=4577 offspring; medical examination; diagnostic criteria 2h 75g OGTT >200mg/dl. Followup for 40 years. | *Epidemiology and natural history/long term risk to baby:* Offspring DIP had higher rates of macrosomia, higher BMI through childhood, and increased T2DM in childhood and early adulthood. | High risk of selection bias as 70% had missing data and moderate risk of analytical bias as limited adjustment for confounders. Funding source not specified. |
| Lindsay 2000b (1965-NA) | United States (Arizona); Pima and Tohono Indian; remote; community based data | Descriptive (quantitative); Retrospective analysis (of longitudinal study) investigate genetic effects of lbw (?"thrifty genotype) on T2DM; n=1608; study data; diagnostic criteria FPG .104mg/dl ot 2h 75g OGTT >200mg/dl. | *Epidemiology and natural history/long term risk to baby:* Maternal DIP posivitely asociated with birthweight and paternal T2DM negatively associated with bw | Low risk of bias. Funding source not specified. |
| Livingston 1993 (1984-1988) | United States (Arizona); Tohono O'odham; remote; clinic based data | Descriptive (quantitative); retrospective cohort investigating prevalence T2DM in pregnancy and incidence GDM; n=1854 women; medical records; diagnostic criteria 1h 50g OPGTT and 3h 100g OGTT if > 145mg/l | *Epidemiology and natural history/prevalence:* Prevalence DIP 5.2%, T2DM 2%, GDM3.2% (crude rates). Authors conclude the high prevalence justifies universal screening in this community. | Moderate risk of outcome assessment bias as dependant on medical records and high analytical bias as no adjustment for confounders. Funding source not specified. |
| Mackerras 1998 (1990-1996) | Australian (Northern Territory); Aboriginal; remote | Intervention; Before-after evaluation of "Strong Women, Strong Babies, Strong Culture" program; n=474 babies; medical records and midwives data; medical diagnosis of DIP. | *Epidemiology and natural history and prevention:* Prevalence GDM only 5.3% (1990-1) and 7.7% (1994-6). Decrease in lbw in intervention communities. | Controlled before-after cluster study therefore no randomisation and high risk of bias. High risk of bias in prevalence data as limited methods description and analysis. Source of funding not specified. |
| Massion 1987 (1985) | United States; Navajo Indian; remote; clinic based data | Measurement; Prospective cohort to evaluate the efficacy of O'Sullivan screening test vs risk factor analysis; n=181; medical records and examination; diagnostic criteria 1h 50g OGTT then 3h 100g OGTT if over 7.2mmol/L | *Efficacy and cost:* GDM 6.1% crude rate. Screening test efficacy at 28-32 weeks had ppv0.25 sensitivity.80 using 7.2mmol/L cutoff after 50g OGTT, which is significantly more sensitive than risk factor analysis alone. Authors recommend universal screening in high risk population. | Low risk of bias. Source of funding not specified. |
| McCance 1994 (1940-1972) | United States (Arizona); Pima Indian; remote reservation; community based study data | Descriptive (quantitative); retrospective cohort (of longitudinal study) investigating prevalence T2DM according to birthweight and exposure to DIP; n=1179 offspring; medical examination from longitudinal study; DIP exposure based on medical diagnosis | *Epidemiology and natural history/long term risk to baby:* U-shaped relationship birthweight and T2DM prevalence. OR 10.73 for GDM in mothers of diabetic offspring. | Low risk of bias. Source of funding not specified. |
| McGrath 2007 (1997-2005) | New Zealand (Northland); Maori; rurality unknown; community based data. | Descriptive (quantitative); retrospective cohort to audit the number of women with GDM followed up; n=110; data source and diagnostic criteria not specified (letter only). | *Systems for followup:* GDM incidence 1.7% (crude rate). 32% women followed up had abnormal glucose tolerance. | High risk of selection bias bias and analytical bias as only 60% followed up and limited methods reported (letter). Moderate risk of outcome assessment bias and confounding bias as relies on database for diagnosis and limited adjustment for confounders. Funded by Novo Nordisk. |
| Mendola 1994 | United States; American Indians | Other: Review of risk factors associatd with developmental disability and prevention | *Epidemiology and natural history/risk to baby:* Preventing maternal diabetes has important role in reducing developmental disabilities | Literature review: question, search strategy, inclusion criteria and quality appraisal not reported. Funding source not specified. |
| Mohamed 1998 (1985-1995) | Canada (Ontario); Ojibwa-Cree; unknown rurality; clinic based data | Descriptive (quantitative); retrospective cohort to examine risk and duration of developing T2DM after GDM, and audit of followup rates; n=61; medical records; WHO diagnostic criteria | *Epidemiology andnatural history/risk to mother & systems for followup:* Prevalence GDM 7.9% (crude rate). 70% developed T2DM, average duration 3 years, only 38% received recommended followup. | Moderate risk of outcome assessment bias as relies on medical record diagnosis and high risk of analytical bias as no adjustment or analysis for confounders. Source of funding not specified. |
| Moum 2004 (1989-2000) | United States (Montana and North Dakota); American Indian; mixed; population based data | Descriptive (quantitative); Retrospective cohort to assess DIP trends; n=133991 (M) & 102232 (ND); birth certificates; Diagnostic criteria DIP or GDM on birth certificate | Prevalence DIP | High risk of outcome assessment bias as diagnosis relies on birth certificate. Funded by CDC. |
| Mouratoff 1969 | United States (Alaska); Athabaskan Indian; remote; community based data | Descriptive (quantitative); Cross-sectional study to investigate glucose tolerance; n=8 pregnant women; medical examination; diagnostic criteria FPG followed by 100g OGTT and 1+2h samples, andmore detailed test in 10 months if FPG >100mg/100ml or 1h > 180mg/100ml or 2h >140mg/100ml. | *Epidemiology and natural history/Prevalence:* 0/8 pregnant women. | High risk of selection bias as small sample and no sample size calculation and moderate risk of bias from confounding. Source of funding not specified. |
| Muller 2010 | United States (Arizona); Pima Indian; remote; community based data | Descriptive (quantitative); Longitudinal/case control study to test genetic susceptiability to T2DM; n=3501 100% Pima Indian heritage, 3723 mimxed heritage, 486 Old Order Amish; Study data; WHO diagnostic criteria. | *Epidemiology and natural history:* Genetic variants in MBL2 contribute to T2DM susceptibility in Native Americans and Old Order Amish. | Low risk of bias. Source of funding not specified. |
| Murphy 1991 (1987-88) | United States (Alaska); Yup'ik Eskimo; remote; community based data | Descriptive (quantitative); retrospective cohort to estimate DIP prevalence; n=873 births; hospital birth data and registry; O'Sullivan and WHO diagnostic criteria | Prevalence T2DM and DIP | Moderate risk of outcome assessment bias as reliant on medical records and registry. Source of funding not specified. |
| Murphy 1993 (1987-1988) | United States (Alaska); Yup'ik Eskimo; remote; community based data | Descriptive (quantitative); retrospective cohort to estimate DIP and T2DM prevalence; n=630 births; hospital birth data and registry; 1h 50g )GTT >7.8mmol/L | *Epidemiology and natural history/prevalence:* Prevalence GDM 5.8% crude and T2DM 0.3% crude. | Moderate risk of outcome assessment bias as reliant on medical records and registry. Source of funding not specified. |
| Murphy 2008 | United States: Native American | Intervention: prospective register (time series) to evaluate the effect of a breastfeeding intervention on T2DM;n=>6000 women; | *Other/Prevention:* BF rates increased. | High risk of bias as no control group, just interrupted time series to measure changes in rates. Source of funding not specified. |
| Naylor 2003 | United States: Alaska Natives | Other: Review of diabetes | *Epidemiology and natural history/prevalence & prevention:* High GDM rates in some regions but appeared to decline after a nutiritonal intervention. | Question and search strategy defined but no risk of bias appraisal reported. Source of funding not specified. |
| Nelson 1998 (1983-1996) | United States (Arizona); Pima Indian; remote; community based data | Descriptive (quantitative); cross-sectional study investigating association between DIP and renal disease; n=502 people with T2DM; examination diagnostic criteria 2h 75g OGTT | *Epidemiology and natural history/long term risk to baby:* Intrauterine exposure to DIP an independent risk factor for renal disease in offspring | Low risk of bias. Source of funding not specified. |
| Nelson 1998b | United States (Arizona); Pima Indian; remote; community based data | Descriptive (quantitative); cross-sectional study investigating association between birthweight and renal disease; n=308 people with T2DM and known birthweight; examination diagnostic criteria 2h 75g OGTT | *Epidemiology and natural history/long term risk to baby:* DIP the major risk factor (not birthweight) in impaired renal function in hbw offspring, but u-shaped curve. | Low risk of bias. Source of funding not specified. |
| Neufeld 2006 | Canada (Manitoba); First Nations; remote;community based | Descriptive (qualitative); Interviews with mothers and grandmothers about perceptions of causes of DIP; n=28 | *Preferences and Values:* Sugar, processed food, lack of exercise, weight gain perceived as causes of DIP. Mothers emphasized stress. Suggested increased fresh food. | Methods appear appropriate and conclusions appear justified. Source of funding not specified. |
| Nicolle 1996 (1989-92) | Canada (Manitoba); First Nations; unknown population; clinic based data | Descriptive (quantitative); retrospective cohort to assess impact of acute pyelonephritis and causes;n=838 patients with pyelonephritis; | *Epidemiology and natural history/Risk to health of mother in pregnancy:*High rates of hospitalization in Native American women with DIP | Moderate risk of outcome assessment bias and confounding as relies on ICD codes and analysis for some confounders only. Funding source not specified. |
| O'Dea 2007 | Australia; Aboriginal and Torres Strait Islander | Other: Expert opinion about strategies to address | *Other/prevention:* Recommends improved DIP control and promoting breastfeeding for 2-3 months to reduce T2DM | NA: opinion only. Source of funding not specified. |
| Osgood 2011 (1956-2006) | Canada (Saskatchewan); First Nations; mixed; population based data. | Descriptive (quantitative); retrospective cohort to model inter- and intra-generational effect of GDM on the T2DM epidemic; whole population; health registry data; diagnosis recorded in database. | *Epidemiology and natural history (Long term risks to infant):* GDM may be repsonsible for 19-30% T2DM cases among First Nation Saskatchewan people and only 6% other Saskatchewan people. | Moderate risk of outcome assessment bias as relies on database reporting. Funded by Saskatchewan Health Research Foundation. |
| Oster 2009 (2003-7) | Canada (Alberta); First Nations; mixed; community based data | Descriptive (quantitative); Prospective cohort to investigate DM amongst mixed blood populations; n=3148 (2111 women); 3 screening program databases; diagnostic criteria FPG >6.9 or RPG >11.0 or A1c >7%. | *Epidemiology and natural history/prevalence:* Prevalence GDM 9%. Full blooded First Nations people had highest risk and mixed blood people had intermediate risk (higher than general population). | Moderate risk of bias from confounding. Funded by Canadian Institute of Health Research. |
| Patel 1989 (1985-87) | Australia (Northern Territory); Aboriginal; remote; unclear | Other: Review of GDM screening and recommendations | *Epidemiology and natural history/prevalence & screening practice:* Cites unavailable report of 3% GDM prior to introduction of universal screening, and 12% Incidence GDM following universal screening (crude rates). T2DM in pregnancy 1.6% crude. | High risk of bias as based on literature review and sources not available for appraisal. Review question, search strategy, inclusion criteria and ROB appraisal not described. Source of funding not specified. |
| Pettitt 1980 (1965-89) | United States (Arizona); Pima Indian; remote; community based data | Descriptive (Quantitative); prospective cohort/longitudinal study to investigate maternal and infant complications of DIP; n=811 pregnancies; study data; diagnostic criteria 2h 75g OGTT >11.1 (WHO). | *Epidemiology and natural history/risk to mother and baby:* Increased risk perinatal mortality, macrosomia, toxemia, an caesarean section directly associated with glucose concentration and predictive of future development of T2DM. | Low risk of bias. Source of funding not specified. |
| Pettitt 1983 (1965-89) | United States (Arizona); Pima Indian; remote; community based data | Descriptive (Quantitative); prospective cohort/longitudinal study to investigate long term effects of DIP on obesity in offspring; n=1935 offspring; study data; diagnostic criteria 2h 75g OGTT >11.1 (WHO). | *Epidemiology and natural history/long term risk to baby:* Offspring of mothers with DIP most likely to be obese. Offspring of women who never developed T2DM or DIP least likely to be obese. | Low risk of bias. Source of funding not specified. |
| Pettitt 1987 (1965-82) | United States (Arizona); Pima Indian; remote; community based data | Descriptive (quantitative); prospective cohort to investigate influence of DIP and birthweight on obesity in offspring; n=112 offspring; study data; 2h 75g OGTT >11.1 (WHO) | *Epidemiology and natural history/long term risk to baby:* Offspring of mothers with DIP most likely to be obese, independent of birthweight. | Low risk of bias. Source of funding not specified. |
| Pettitt 1988 (1965-?) | United States (Arizona); Pima Indian; remote; community based data | Descriptive (quantitative); prospective cohort to investigate susceptability of DIP offspring to T2DM;n=1064 offspring; 2h 75g OGTT >11.1 (WHO) | *Epidemiology and natural history/long term risk to baby:* Offspring exposed to DIP have 45% risk of T2DM at age 20-24, compared with 8.6% offspring of "prediabetic" women who developed T2DM after pregnancy, and 1.4% offspring of nondiabetic women. Independant of paternal T2DM and BMI. Suggest intrauterine environment an important determinant. | Low risk of bias. Source of funding not specified. |
| Pettitt 1991 (1965-1989) | United States (Arizona); Pima Indian; remote; community based data | Descriptive (quantitative); prospective cohort to investigate glucose tolerance in offspring of women with abnormal glucose tolerance in pregnancy (not GDM); n=552 offspring; study data; 2h 75 OGTT 11.1 | *Epidemiology and natural history/long term risk to baby:* Offspring of women with abnormal glucose (but not GDM) have impaired glucose tolerance and increased risk of DIP. | Moderate risk of bias from confounding. Funded by American Diabetes Association. |
| Pettitt 1993 (1965-?) | United States (Arizona); Pima Indian; remote; community based data | Descriptive (quantitative); prospective cohort to investigate long terms effects of DIP on obesity in offspring; n=1112; study data; 2h 75g OGTT >11.1 (WHO) | *Epidemiology and natural history/long term risk to baby:* Offspring of women with DIP have a higehr risk of obesity, higher glucose levels and more T2DM. | Low risk of bias. Source of funding not specified. |
| Pettitt 1994 (1992) | United States (Arizona); Pima Indian; remote; community based data. | Measurement: prospective cohort to compare efficacy of WHO and National Diabetes Data Group GDM screening criteria; n=127; study data; 2h 75 OGTT and NDDG criteria. | *Screening test efficacy and cost:* One step WHO test has higher sensitivity and is less cumbersome than NDDG criteria (WHO specificity 93% and NDDG only 18% sensitivity compared to WHO criteria). Who criteria more predictive of poor outcomes. | High risk of selection bias as only 35.9% eligible included and selection not described. Moderate risk of confounding. Source of dunign not specified. |
| Pettitt 1996 (1965-95) | United States (Arizona); Pima Indian; remote; community based data | Descriptive (quantitative); prospective cohort to determine if non-pregnant women with glucose intolerance also have high risk of developing T2DM; n=317; study data; 2h 75g OGTT >11.1 (WHO); followup less than 10 years if entering study after 1985. | *Epidemiology and natural history/long term risk to women:* Non-pregnant women with impaired glucose tolerance have a higher risk of developing T2DM than pregnant women with glucose intolerance. | Low risk of bias. Source of funding not specified. |
| Pettitt 1998 (1965-?) | United States (Arizona); Pima Indian; remote; community based data | Descriptive (quantitative); prospective cohort to determine long terms effects of DIP, birthweight and breastfeeding; n=1536 offspring; study data; 2h 75g OGTT >11.1 (WHO) | *Epidemiology and natural history/long term risk to baby and prevention:* DIP is a major risk factor for T2DM and hyperglycaemia in offspring. LBW increases risk further. BF decreases risk. | Moderate risk of bias from confounding. Source of funding not specified. |
| Pratley 1998 | United States (Arizona); Pima Indians; remote; community based data. | Review of evidence for genetic and environmental interactions in the pathogenesis of T2DM. | *Epidemiology and natural history (Long term risks to infant):* Genetic and environmental factors (including exposure to diabetic intra-uterine environment) associated with an increased risk of T2DM. | Review only. No description of search strategy or assessment of study quality. |
| Richards 2012 | United States (Northern Plains); Lakota; remote; community. | Randomised controlled trial of community-based participatory research to improve preconceptual health and cultural knowledge. | *Other (Primary Prevention):* Intervention group showed a signficantly higher rate of knowledge of diabetes and obesity after the intervention than the control group. | Moderate risk of bias. >20% attrition not included in outcome analysis. |
| Rith-Najarian 1996 (1990-1992) | United States (Minnesota): Chippewa; remote; community based data | Descriptive (quantitative); retrospective cohort to determine risk of GDM and screening rates; n=684 pregnancies; local health service registry and outpatient records; 1h 50g OGTT >140mg/gl and then O'Sullivan diagnosis. | *Epidemiology and natural history/prevalence & screening practice:* Incidence GDM 5.8% crude and T2DM in pregnancy 1% crude. 82% women screened. | Moderate risk of outcome assessment bias as relies on medical records and high risk of confounding as no adjustment. >10% data excluded. Source of funding not specified. |
| Roberts 1990 (1968-1987) | New Zealand; Maori; mixed; clinic based data. | Descriptive (quantitative); retrospective cohort to analyse perinatal outcomes among women with diabetes over 20 years; all clinic; hospital data; diagnosis varied but largely O'Sullivan's criteria using 100g OGTT. | *Epidemiology and natural history (risks to women during pregnancy and birth):* Women with DIP had an increased risk of caesarean section. PMR has decreased signficantly from 1968 (6.7%) to 1987 (0.5%) among women with GDM. | Unclear if any adjustment or controlling for confounders (Moderate ROB). |
| Rodrigues 1999 (1995-1997) | Canada (James Bay, Quebec); Cree; remote; clinic based | Descriptive (quantitative); cross-sectional study to determine prevalence of GDM; n=703 pregnancies; birth records; NDDG criteria 1h 50g OGTT and 3h 100g OGTT >7.2mmol/L | *Epidemiology and natural history/prevalence:* Incidence GDM 12.8% crude, which is twice as high as the non-Aboriginal population. | Moderate risk of outcome assessment bias as relies on medical records and moderate risk of bias from confounding. Source of funding not specified. |
| Rodrigues 1999b (1995-6) | Canada (James Bay, Quebec); Cree; remote; clinic based | Descriptive (quantitative); cross-sectional study to determine interaction between body weight and ethnicity and risk of GDM; n=796 Cree, 8506 non-Cree; birth database and maternal recall (subset Cree women only); NDDG criteria 1h 50g OGTT and 3h 100g OGTT >7.2mmol/L | *Epidemiology and natural history/risk factors:* Normal weight Cree women hads same risk of GDM as non-Cree normal weight women, but overweight Cree women had a higher risk than overweight non-Cree women. | High risk of selection bias as only 59% records had complete data and moderate risk of outcome assessment bias as relies on medical records. Source of funding not specified. |
| Rodrigues 2000 (1995-6) | Canada (James Bay, Quebec); Cree; remote; clinic based | Descriptive (quantitative); cross-sectional study to examine independent risk factors for infant macrosomia; n=385 Cree and 5644 non-Cree; birth database; NDDG criteria 1h 50g OGTT and 3h 100g OGTT >7.2mmol/L | *Epidemiology and natural history/risk to baby:* Prevalence macrosomia 34.3% in Cree babies compared to 11.1% general pop, despite controlling for GDM and maternal BMI . Authors suggest it may be due to different treatment strategies. | Moderate risk of outcome assessment bias as relies on birth database. Funded by Canadian Diabetes Association. |
| Rumbold 2011 (2009) | Australia; Aboriginal and Torres Strait Islander; remote; clinic based data. | Descriptive (quantitative); cross-sectional survey of access to antenatal care services; n=535 records in 34 community controlled health clinics; OGCT then OGTT. | *Current screening practice and rates:* 51% pregnant women screened for GDM (rnge 33-78%). Of women who had an abnormal OGCT, 77% had a diagnostic OGTT. | Moderate risk of selection bias as self-selected clnics participated. Funded by Cooperative Research Centre for Aboriginal Health. |
| Salbe 1998 (1991-6) | United States (Arizona); Pima Indian; remote; community based data | Descriptive (quantitative); cross-sectional study to determine effect of DIP on energy expenditure in 5yo offspring; n=88 5yo children; medical examination and tests; 2h 75g OGTT >11.1 mmol | *Epidemiology and natural history/long term risks to baby:* No differences in BMI or exercise expenditure in 5yo offspring | Low risk of bias. Source of funding not specified. |
| Sayers 2009 | International | Other: Review of Indigenous Newborn Care | *Epidemiology and natural history/ risks to baby:* GDM a cause of poor birth outcomes for Indigenous babies | Literature review: question, search strategy, inclusion criteria and ROB appraisal not specified. Source of funding not specified. |
| Scavini 2003 (1999-2002) | United States (New Mexico); Zuni Indian; remote reservation; population based data | Descriptive (quantitative); cross sectional survey to determine if prevalence of DM similar between males and females; n=1503 people over 5 years; Self-report questionnaire and examination; Diagnostic criteria HbA1c >7mmol/L | *Epidemiology and natural history/prevalence:* 20.9% female survey respondents reported a history of GDM. Higher prevalence T2DM amongst femailes (16.7%) than males(9.7%). | High risk of selection bias as only 16% eligible population included and selection process not described, moderate risk of outcome assessment bias and moderate risk of confounding bias. Source of funding not specified. |
| Schaefer 1970 (1958-1968) | Canada; Inuit; mixed; population-based data. | Descriptive (quantitative); retrospective cohort to investigate rates of sugar consumption and birth weights. | *Epidemiology and natural history (risk to infant in pregnancy and birth):* Increased birth weight correlated with increased sugar consumption. | High risk of outcome assessment bias as no DIP diagnosis and high risk confounding bias as no adjustment . |
| Schiff 1999 (1994) | United States (New Mexico); American Indian; mixed; population based data | Descriptive (quantitative); retrospective cohort to investigate risk factors for caesarean birth; n=3645 births; birth certificate data; diagnostic criteria was DIP recorded as birth complication. | *Epidemiology and natural history/risks to mother during pregnancy and birth:* DIP 5.5% crude rate. Caesarean section risk factors include prematurity, LBW, diabetes, hypertension, birth complications | Moderate risk of outcome assessment bias as relies on birth certificate data. Source of funding not specified. |
| Shah 2011 | International;Indigenous. | Systematic review of pregnancy and neonatal outcomes among indigenous women. | *Epidemiology and natural history (risk to infant in pregnancy and birth):* Aboriginal women are at increased risk of adverse pregnancy outcomes. Significantly higher rates of diabetes suggested as a potential implicating factor. | Systematic review with quality appraisal. Low ROB. |
| Shen 2008 | Canada;First Nations | Other: Editorial discussing weight gain during pregnancy | *Other/prevention:* Discusses ethnographic study about weight gain in pregnancy (Willows 2008) which suggests a limited understanding about healthy weight gain, and identified barriers to exercise and healthy food. | NA: editorial |
| Sievers 1976 | United States; American Indian | Other: Standards for diagnosis and management | *Adequate treatment pathways:* General recommendations | NA: standards based on recommendations of a Diabetic Subcommittee. Limited discussion of evidence base and consensus process to develop guidelines. |
| Silva 2006 (1995-2005) | United States (Hawaii); unknown rurality; clinic based data | Descriptive (quantitative); retrospective cohort to determine perinatal outcomes associated with GDM by ethnic group; n=2155 patients with GDM; clinic records; diagnostic criteria based on Coustan (3h OGTT) or 1g .200mg/dl | *Epidemiology and natural history/risk to mother and baby:* Prevalence GDM 3.6%. Native Hawaiin children had 4 times risk of macrosomia compared to other mothers with GDM. | Moderate risk of outcome assessment bias as relies on medical diagnosis and referral. Funded by National Center for Minority Health and Health Disparities, NIH. |
| Simmons 2003 (1999-2001) | Australia (Victoria); Aboriginal; rural; clinic based data | Intervention: Before-after evaluation of integrated community controlled diabetes care program; n=47; medical records; diagnostic criteria HbA1c>9% | *Adequate treatment pathways:* Improved self monitoring, but no significant weight loss | High risk of bias as no control group: process evaluation only. Source of funding not specified. |
| Simmons 2005 (1998-1999) | Australia (Victoria); Aboriginal; rural; clinic based data | Descriptive (quantitative); retrospective cohort to compare antenatal care and obstetric outcomes; n=112 non-Aboriginal (age-matched controls), 29 Aboriginal; medical records; diagnostic criteria 50g OGCT the 75g OGTT if >7.8mmol/L | *Epidemiology and natural history/prevalence:* Prevalence GDM 10.7% (not stated if T2DM included). Aboriginal women had higher rates of GDM, were smaller and were less likely to BF. | Moderate risk of outcome assessment bias as relies on medical records and moderate risk of confounding bias. Funded by Commonwealth Department of Health and Ageing. |
| Simmons 2011 (1991-1994) | New Zealand; Maori; mixed; clinic based data. | Case control study (matched 2:1 on ethnicity and type of diabetes) comparing use of insulin pump therapy with women receiving single dose insulin injections; n=7/30 women receiving insulin pumps were Maori; 2h 75g OGTT ADIPS criteria used. | *Adequate treatment pathways:* Women receiving insulin pump therapy has improved glycaemic control, despite higher weight gain and insulin requirements. Rates of neonatal hypoglycaemia were similar but infants of mothers receiving pump therapy were more likely to be admitted to NICU. | High risk of bias as no randomisation and signficant differences in baseline insulin requirements and weight gain between study arms. |
| Smith-Morris 2005 (1997-2000) | United States (Arizona); Pima Indian; remote; community | Descriptive (qualitative); Ethnographic study to investigate the meaning of risk associated with GDM; n=63; Interviews and quantitative study data | *Preferences and Values:* Confusion over diagnosis amongst both professionals and women. Heterogenous understandings. | Question and methods appear appropriate. Not clear if results address research question and conclusions are justified. Source of funding not specified. |
| Stanley 1985 (1980-82) | Australia (Western Australia); Aboriginal; mixed; population based data | Descriptive (quantitative); Retrospective cohort to investigate rate of congenital malformations amongst babies of mothers who had DIP; n=225 babies; midwvies data collection records; diagnostic criteria based on DIP recorded on form, as well as Aboriginality. | *Epidemiology and natural history/risk to baby:* Prevalence DIP 15.8/1000 (crude). DIP associated with increased risk of congenital abnormalities. | Moderate risk of outcome assessment bias as relies on midwives birth data and moderate risk of confounding. Source of funding not specified. |
| Steinhart 1997 (1983-87) | United States (New Mexico); Navajo Indian; remote; community based data | Descriptive (quantitative); Retrospective identification and then prospective cohort to estimate rate of progression from GDM to T2DM; n=111 women with GDM; medical records and examination; diagnostic criteria 1h 50g OGCT. If >7.2 then 3h 100g OGTT (ACOG guidelines) then WHO standards to followup T2DM screening. Followup for 11 years. | *Epidemiology and natural history/progression to T2DM:* Prevalence GDM 4.6% crude rate.50-70% progressed to T2DM within 11 years | Moderate risk of outcome assessment as relies on medical records for GDM diagnosis and moderate risk of confounding. High risk of attrition bias as 29% did not return for testing. Source of funding not specified. |
| Stephenson 1993 | Australia (New South Wales); Aboriginal; | Other: Opinion about strategies to address T2DM | *Other/prevention:* Authors argue broader community based environmental interventions are required to address DM. | NA: opinion |
| Stone 2002 (1996) | Australia (Victoria); Aboriginal; mixed rural and urban; population based data | Descriptive (quantitative); retrospective cohort to measure incidence, risk factors and outcomes of DIP; n=59962 births; linked birth and hospital discharge data; diagnostic criteria based on diagnosis on record | *Epidemiology and natural history/prevalence:* DIP prevalence DIP 4.3% Aboriginal and 3.6% non-Aboriginal crude rate. | Moderate risk of outcome assessment bias as relies on database and moderate risk of confounding. Source of funding not specified. |
| Sugarman 1989 (1983-87) | United States (New Mexico); Navajo Indian; remote; community based data | Descriptive (quantitative); retrospective cohort to measure prevalence of DIP; n=4094 births; Linked MCH records; birth register and ICD discharge codes; medical diagnosis recorded on database | *Epidemiology and natural history/prevalence:* Prevalence GDM 3.4% and DIP 4.6% crude rate. | Moderate risk of outcome assessment bias as relies on database record and medical diagnosis and moderate risk of confounding. Funded by the Indian Health Service Research Program. |
| Templeton 2008 (2001-6) | Australian; Aboriginal and Torres Strait Islander; mixed; population based | Descriptive (quantitative); retrospective cohort to describe the prevalence of GDM; n=12403 births; Linked hospital and diabetes databases; diagnostic criteria diabetes recorded in dataset | *Epidemiology and natural history/prevalence:* Incidence GDM 4.8% crude rate; 12.3% amongst 35-39 yo women (age adjusted). | Moderate risk of outcome assessment bias as relies on database and moderate risk of confounding bias. Funded by Commonwealth Government. |
| Thomson 1990 (1982-1986) | Canada (Ontario); Native Indians; mixed; population based data | Descriptive (quantitative); retrospective cohort to investigate if birthweight is increasing; n=4724 Indian births and 206779 non-Indian births; birth registry data; diagnostic criteria diabetes recorded on dataset. | *Epidemiology and natural history/risk to baby:* Macrosomia 50% more frequent in Indian than non-Indian babies (RR1.47, 95% CI1.35-1.59). Authors hypthesise this is due to high rates of glucose intolerance. | Moderate risk of outcome assessment bias as relies on database and moderate risk of confounding bias. Funded by Ministry of Health. |
| Vallianatos 2006 | Canada (James Bay); Cree; remote; interview data | Descriptive qualitative study of womens beliefs and practices about weight gain during pregnancy and postpartum; n=30. | *Preferences or values:* Women expressed concern about limiting weight gain in pregnancy and recognised importance of losing weight postpartum, but discussed a number of barriers including: lack of energy, isolation at home, comfort eating, lack of knowledge, cultural beliefs, lack of community support and services. | Moderate risk of bias. |
| Whincup 2008 | Indigenous; international. | Systematic review of evidence for association between birthweight and T2DM, with subgroup analysis by ethnicity; n=30 studies (all indigenous studies already included in this review) | *Epidemiology and natural history (long term risks to infants):* U-shaped association between T2DM and birthweight among Pima Indian and Native Indian people. | Moderate risk of bias. |
| Williams 1999 (1987-95) | United States (Washington State); Native Americans;mixed; population based data | Descriptive (quantitative); Retrospective cohort to investigate association of maternal birthweight and risk of future GDM; n=7456 NA; 2156 W; 6359 AA;6496 H; birth registry data; criteria based on diabetes recorded in register | *Epidemiology and natural history/risk factors for developing GDM:* GDM incidence 2.7%.Native American women with bw <2000g 3.1 times more likely to have GDM (White women 1.7 times more likely). | Moderate risk of selection bias (>10% missing data) and outcome assessment bias as relies on GDM record in registry. Funded by Maternal and Child Health Bureau. |
| World Health Organisation 1992 (1976-1991) | International | Other: Review of prevalence of T2DM amongst women of childbearing age. Includes popualtion survey data which uses WHO diagnostic criteria 2h 75g OGTT >7.8. | *Epidemiology and natural history/prevalence T2DM:* Prevalence amongst Pima Indian women the highest in the world (22.5% crude rate). | Question, search strategy and inclusion criteria clear however risk of bias appraisal not described. Source of funding not specified. |
| Wu 2005 (2003) | United States (Guam); Chammorro; urban; population based data | Descriptive (quantitative); Cross-sectional survey to investigate diabetes prevalence and risk factors as formative evaluation for an intervention; n=228; survey data; diagnostic criteria based on self-report | *Epidemiology and natural history/prevalence:* GDM Prevalence 4.7% (n=6). | Moderate risk of outcome assessment bias from self-report and moderate risk of confounding. Source of funding not specified. |
| Yapa 2000 (1994-5) | New Zealand (Auckland); Maori; rurality not clear; clinic based data | Descriptive (quantitative); Retrospective cohort to audit GDM screening rates and investigate characteristics of people receiving screening; n=4885 medical records; diagnostic criteria 1h 50g OGTT 24-28 weeks, then 2h 75g OGTT if >7.8. | *Epidemiology and natural history/prevalence & screening practice and rates:* GDM incidence 5.7% crude rate (7.9% if adjusted for OGTT attendance). Only 47.3% of women screened. | Moderate risk of outcome assessment bias as relies on medical records and moderate risk of confounding. Funded by South Auckland diabetes project. |
| Young 2002 | Canada (Manitoba); First Nations; remote; clinic based data | Descriptive (quantitative); Case control study to investigate prenatal and infant risk factors for T2DM in children; n=46 cases & 92 age/sex matched controls; survey data; diagnostic criteria based on self-report questionnaires | *Adequate treatment pathways:*DIP a significant risk factor for T2DM, so recommends early screening and BF to decrease risk. | Selection bias unclear. Moderate risk of outcome assessment bias from self-report and subject to recall bias and moderate risk of confounding bias. Funded by the Canadian Diabetes Association. |
| Yue 1996 | Australia (New South Wales); Aboriginal; rurality unclear; clinic based data | Descriptive (quantitative); retrospective chort to investigate GDM prevalence; n=3817; linked diabetes and antenatal clinic data; diagnostic criteria 1h 50g GCT then 2h 75g OGTT (ADIPS criteria) | *Epidemiology and natural history/prevalence:* GDM 10.1% (crude rate) amongst Aboriginal women (3% Anglo-celtic women), and shorter progression to T2DM, which authors argue supports the "underwater volcano" hypothesis of T2DM amongst Aboriginal people. | Sampling unclear. Moderate risk of outcome assessment bias and confounding bias. Source of funding not specified. |
